# Novel 3-Acetyl-2,5-disubstituted-1,3,4-oxadiazolines: Synthesis and Biological Activity

**DOI:** 10.3390/molecules25245844

**Published:** 2020-12-10

**Authors:** Kinga Paruch, Łukasz Popiołek, Anna Biernasiuk, Anna Hordyjewska, Anna Malm, Monika Wujec

**Affiliations:** 1 Chair and Department of Organic Chemistry, Faculty of Pharmacy, Medical University of Lublin, 4A Chodźki Street, 20-093 Lublin, Poland; lukasz.popiolek@umlub.pl (Ł.P.); monika.wujec@umlub.pl (M.W.); 2 Chair and Department of Pharmaceutical Microbiology, Faculty of Pharmacy, Medical University of Lublin, 1 Chodźki Street, 20-093 Lublin, Poland; anna.biernasiuk@umlub.pl (A.B.); anna.malm@umlub.pl (A.M.); 3 Chair and Department of Medicinal Chemistry, Faculty of Medical Dentistry, Medical University of Lublin, 4A Chodźki Street, 20-093 Lublin, Poland; anna.hordyjewska@umlub.pl

**Keywords:** 1,3,4-oxadiazoline derivatives, antimicrobial activity, hydrazide–hydrazones

## Abstract

The aim of our study was the two-stage synthesis of 1,3,4-oxadiazole derivatives. The first step was the synthesis of hydrazide–hydrazones from 3-methyl-4-nitrobenzhydrazide and the corresponding substituted aromatic aldehydes. Then, the synthesized hydrazide–hydrazones were cyclized with acetic anhydride to obtain new 3-acetyl-2,3-disubstituted-1,3,4-oxadiazolines. All of obtained compounds were tested in in vitro assays to establish their potential antimicrobial activity and cytotoxicity. Our results indicated that few of the newly synthesized compounds had some antimicrobial activity, mainly compounds **20** and **37** towards all used reference bacterial strains (except *Klebsiella pneumoniae*, *Proteus mirabilis*, and *Pseudomonas aeruginosa*) and fungi. These substances showed a strong or powerful bactericidal effect, especially against *Staphylococcus* spp. belonging to Gram-positive bacteria. Compound **37** was active against *Staphylococcus epidermidis* at minimal inhibitory concentration (MIC) = 0.48 µg/mL and was characterized by low cytotoxicity. This compound possessed quinolin-4-yl substituent in the second position of 1,3,4-oxadiazole ring and 3-methyl-4-nitrophenyl in position 5. High effectiveness and safety of these derivatives make them promising candidates as antimicrobial agents. Whereas the compound **20** with the 5-iodofurane substituent in position 2 of the 1,3,4-oxadiazole ring showed the greatest activity against *S. epidermidis* at MIC = 1.95 µg/mL.

## 1. Introduction

Antibiotic resistance has now become a global problem [1]. Countries around the world are struggling with it, which is why the fight against microbial resistance has been identified as a priority issue in the field of public health by several major global organizations and agencies, i.e., the European Food Safety Authority, the European Medicines Agency, and the European Prevention Office and Disease Control [1]. Thus, the synthesis of new compounds with potential antimicrobial activity is still a major challenge in science today.

The largest number of acute infections described in the literature are caused by *Staphylococcus aureus* and *Staphylococcus epidermidis*. It is believed that these etiologic factors most often cause life-threatening infections during inpatient treatment and in palliative care centers [2]. An additional difficulty in controlling these pathogens is their resistance to β-lactam antibiotics and even methicillin. These types of strains are called: methicillin-resistant *Staphylococcus epidermidis* (MRSE) and methicillin-resistant *Staphylococcus aureus* (MRSA) [3]. They cause infection mainly in people with low immunity. These bacterial strains possess a unique ability to adhere to surfaces without the receptors, only through the production of mucus. Due to this patients with any foreign bodies (i.e., patients after recent surgery with implanted artificial valves or implants, catheterized, intubated, or dialyzed) are extremely susceptible to the risk of infection [4,5]. Treatment of infections caused by these microorganisms is particularly difficult because the source of infection is their natural reservoir, i.e., man. As a result of this many species of staphylococci species are part of our natural microflora covering the skin, throat or the atrium of the nose [6,7].

**Figure 1:**
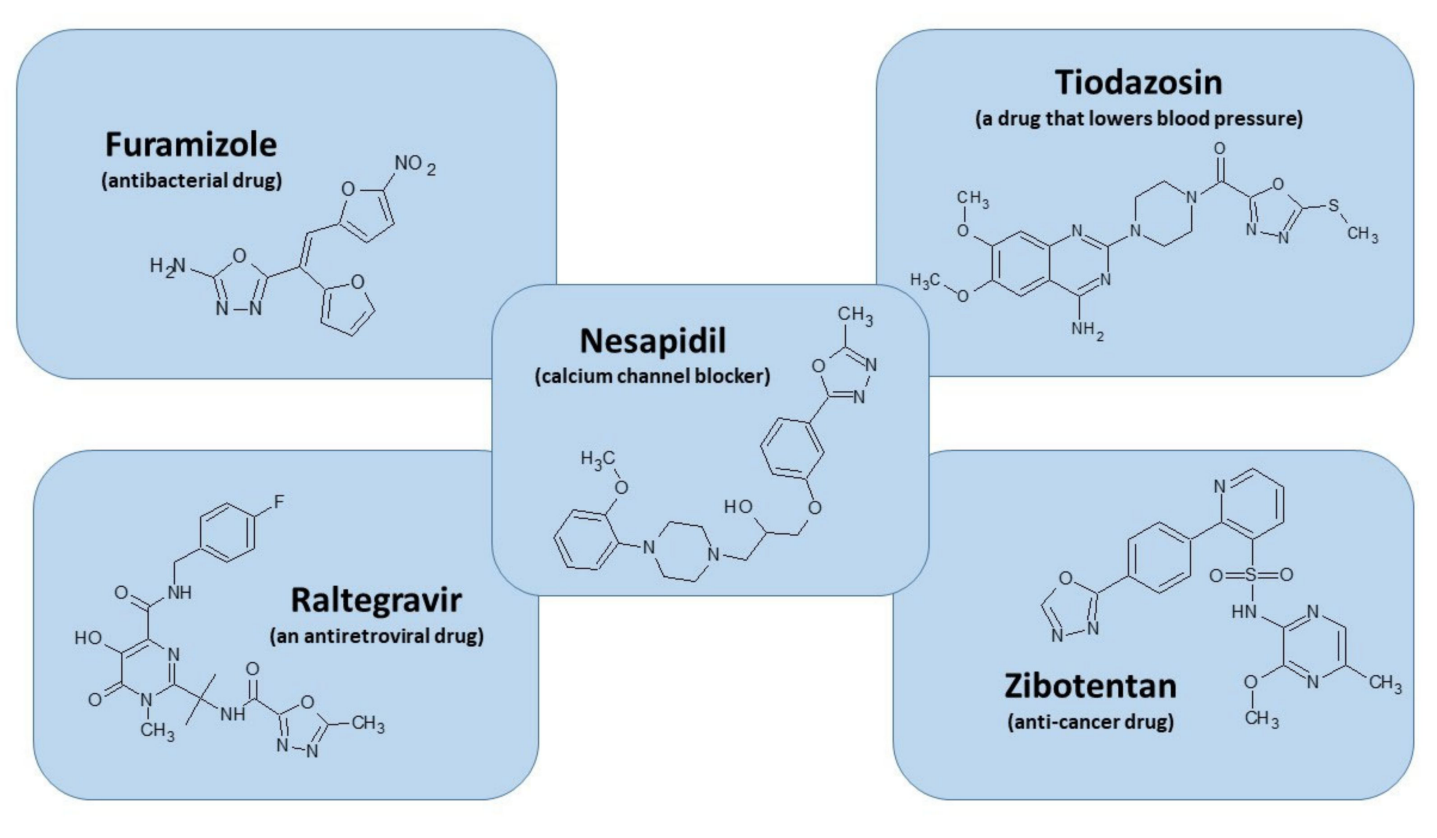
Commonly used medicines bearing 1,3,4-oxadiazole system.

Recent research conducted in our group has revealed that 3-acetyl-2,5-disubstituted-1,3,4-oxadiazolines show significant bioactivity [8,9]. These derivatives have a broad spectrum of activity mainly: antibacterial [10,11,12,13,14,15], antifungal [16,17,18,19], antitubercular [20,21,22], antiprotozoal [23,24], anti-cancer [25,26,27,28], antioxidant [29,30]. They also can act as monoamine oxidase inhibitors [31,32,33,34]. In addition, commonly used drugs, such as Furamizole [35], Nesapidil [36], Raltegravir [37], Tiodazosin [38,39], and Zibotentan [40,41] contain 1,3,4-oxadiazole system (Figure 1).

The 3-acetyl-1,3,4-oxadiazolines are usually obtained as a result of the cyclization reaction of the corresponding hydrazones in an acetic anhydride medium [12,30,42,43,44,45].

The mechanism of the antibacterial action of 1,3,4-oxadiazoles may result from the presence of the –N=CO group in their structure and the transcription of genes involved in biofilm formation, such as SARA (regulator of gene expression), icaA (polysaccharide intercellular adhesion (PIA) or polymeric *N*-acetylglucosamine (PNAG) production), spa (surface protein for bacterial aggregation), fnbA (fibronectin-binding protein A) and fnbB (fibronectin-binding protein B). The gene necessary for biofilm formation in *S. aureus* as well as the main global regulator of the diverse range of virulence determinants in *S. aureus* [9,10].

The 3-acetyl-2,5-disubstituted-1,3,4-oxadizole derivatives seems to be promising antimicrobial agents especially because the seriousness of the growing threat from bacteria fully indicates the urgent need to search for new chemotherapeutics that are stable to the resistance mechanisms created by microorganisms.

## 2. Results and Discussion

### 2.1. Chemistry

**Scheme 1:**
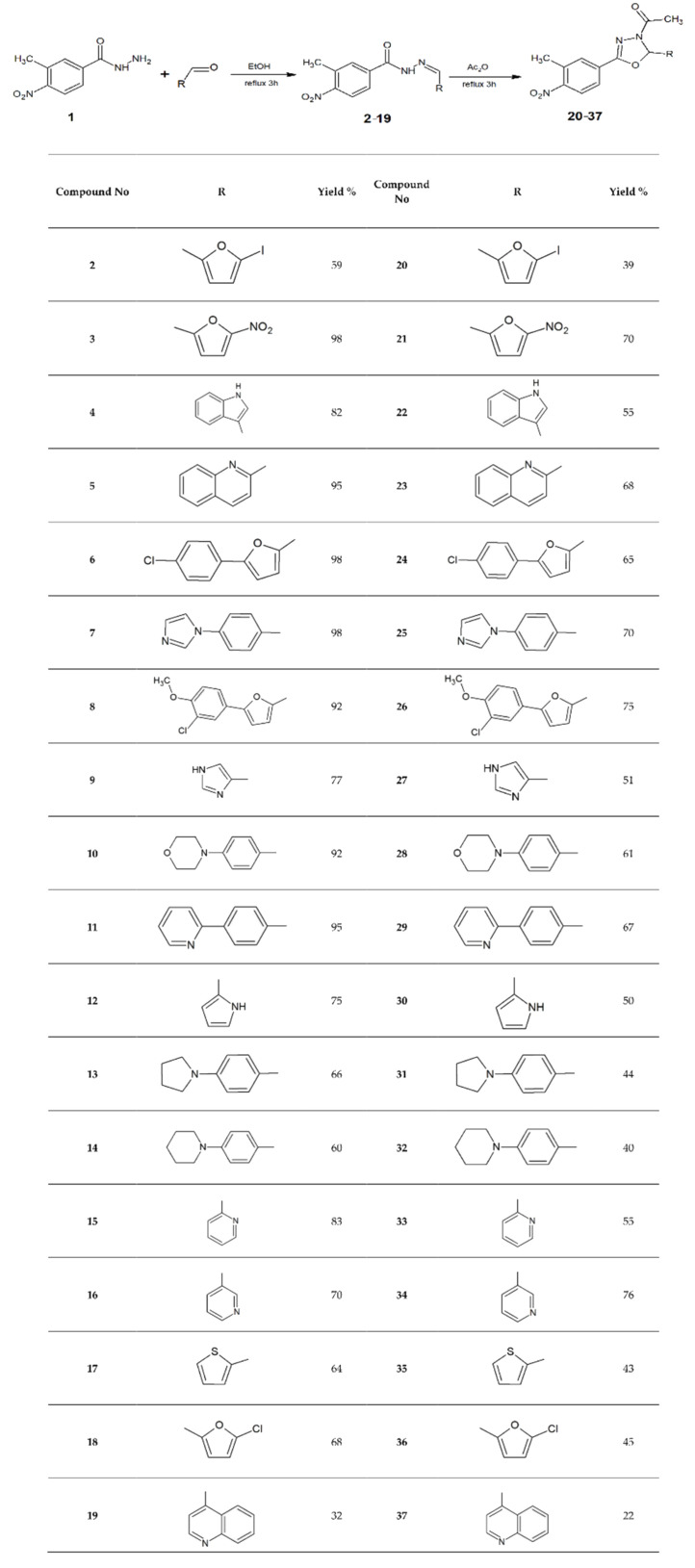
Reaction leading to new hydrazide–hydrazones and 3-acetyl-2,5-disubstituted-1,3,4-oxadiazoline derivatives.

For this research, we synthesized new hydrazide–hydrazones (**2**–**19**) and 3-acetyl-2,5-disubstituted 1,3,4-oxadiazoline derivatives (**20**–**37**) with the use of the cyclization reaction of appropriate hydrazide–hydrazones (**2**–**19**) with neat acetic anhydride (Scheme 1).

Novel hydrazide–hydrazones (**2**–**19**) were obtained in good yields (59–98%), but 3-acetyl-2,5-disubstituted-1,3,4-oxadiazoline (**20**–**37**) were obtained in lower yields (10–35%). Synthesized compounds are stable solids and could be dissolved in DMSO at room temperature. The successful synthesis of novel derivatives was confirmed by analyzing observed signals on ^1^H and ^13^C NMR spectra.

Hydrazide–hydrazones (**2**–**19**) possessed the following characteristic signals on the ^1^H NMR spectra, which confirmed the successful conduction of condensation reactions: singlet for the NH group in the range of 11.73–12.91 ppm and signal for the =CH group at δ 8.23–9.11 ppm. Signals for other fragments of synthesized molecules (**2**–**19**) were observed at expected values of chemical shift. In the case of ^13^C NMR spectra, signals for =CH and C=O groups appeared in the range of δ 137.11–138.61 ppm and 161.18–185.41 ppm, respectively. Additionally, we observed characteristic signals for =CH and C=O groups at expected regions in the FT-IR spectra.

On the other hand, for 3-acetyl-2,5-disubstituted-1,3,4-oxadiazoline derivatives (**20**–**37**) in the ^1^H NMR, the singlet signal for the CH group of 1,3,4-oxadiazoline was observed at δ 7.15–10.01 ppm and for the acetyl substituent for the methyl group, it appeared at δ 1.93–2.57 ppm. Signals for the CH group and the carbon atom of 1,3,4-oxadiazoline in the ^13^C NMR spectra were found at δ 88.41–125.08 ppm and δ 150.83–155.08 ppm, respectively. Similarly, the signal for the methyl group of the acetyl substituent appeared around 20 ppm. Additionally, we observed characteristic signals for C=O, C=N and C-OC bonds at the expected values in FT-IR spectra.

The reaction leading to new 2,5-disubstituted 3-acetyl-1,3,4-oxadiazoline derivatives (**2**–**37**) is presented in Scheme 1.

### 2.2. Microbiology

Synthesized hydrazide–hydrazones (**2**–**19**) and 1,3,4-oxadiazoline derivatives (**20**–**37**) were screened in vitro against a panel of Gram-positive bacteria, Gram-negative bacteria and fungi from *Candida* spp. and subjected to cytotoxicity analysis.

Table 1 and Table 2 present compounds, which displayed some antimicrobial activity. Most of these substances showed antibacterial effect against reference Gram-positive bacteria. Among them, **2**, **20**, and **37** indicated the highest antibacterial activity with minimal inhibitory concentrations (MICs) ranged from 0.48 µg/mL to 500 µg/mL and minimal bactericidal concentrations (MBCs) from 0.48 µg/mL to 1000 µg/mL towards all Gram-positive bacteria. These compounds showed strong activity with a bactericidal effect against staphylococci, especially towards *Staphylococcus epidermidis* American Type Culture Collection (ATCC) 12228 (MIC = MBC = 0.48–15.62 µg/mL and MBC/MIC = 1–2) and good or moderate bactericidal activity towards other Gram-positive bacteria (MIC = 31.25–500 µg/mL, MBC = 31.25–1000 µg/mL and MBC/MIC = 1–4). The best active compound **37** had quinolin-4-yl substituent in the second position of oxadiazole ring and 3-methyl-4-nitrophenyl in position 5. While compounds **2** and **20** had the same substituent 5-iodofuran but compound **2** is hydrazide-hydrazone and compound **20** is 1,3,4-oxadiazole derivative (Table 1 and Table 2).

The substances **2**, **14**–**16**, **24**, and **32** also showed antimicrobial effect against all Gram-positive bacteria, but much weaker or almost no activity with MIC = 62.5–1000 µg/mL, MBC = 125–>2000 µg/mL). *S. epidermidis* ATCC 12228 was sensitive to all tested compounds (MIC = 0.48–1000 µg/mL and MBC = 0.48–>2000 µg/mL), particularly to substances **2**, **20**, **21**, **30**, and **37** (very strong, bactericidal effect with MIC = 0.48–7.81 µg/mL). The activity of compound **37** was more than 8 times greater than that of nitrofurantoin and compound **20** indicated activity more than two times greater. Other substances showed moderate and mild activity or were inactive towards Gram-positive bacteria (Table 1 and Table 2).

The activity of compounds **20** and **37** against *S. aureus* ATCC 25923 and *S. aureus* ATCC 6538 strains ranged from 7.81–15.62 µg/mL, which was a value comparable to the activity of nitrofurantoin and even for compound **37** twice as high (Table 1 and Table 2).

In the case of Gram-negative bacteria, *Bordetella bronchiseptica* ATCC 4617 was the most sensitive to studied compounds (**6**, **12**, **13**, **19**, **20**, **22**, **24**, **27**, **30**–**32**, and **37**). Moreover, substance **20** indicated the highest activity towards this bacteria and rods from *Enterobacterales* family with good, bactericidal effect (MIC = 62.5–125 µg/mL, MBC = 125–500 µg/mL and MBC/MIC = 2–4). Compound **20** showed also mild, bactericidal activity towards *Pseudomonas aeruginosa* ATCC 9027 (MIC = 1000 µg/mL and MBC = 2000 µg/mL, MBC/MIC = 2). Moreover, compounds **24** and **37** exhibited moderate or mild effect against Gram-negative bacteria and no activity towards *Klebsiella pneumoniae* ATCC 13883, *Proteus mirabilis* ATCC 12453, and *P. aeruginosa* ATCC 9027(Table 1 and Table 2).

The results presented in Table 1 and Table 2 also indicated some antifungal effect of tested compounds against yeasts belonging to reference *Candida* spp. Among studied substances, derivatives numbered **6**, **12**, **13**, **19**, **20**, **24**, **31**, **32**, **34**, and **37** showed anticandidal activity towards all reference strains. Derivative **20** had good fungicidal effect with MIC = 31.25–125 µg/mL and MFC = 62.5–500 µg/mL (MFC/MIC = 2–4). Substances **22**, **24**, and **37** indicated varied, both fungicidal and fungistatic activity (MIC = 15.62–500 µg/mL, MFC = 15.62–2000 µg/mL and MFC/MIC = 1–16). In the case of compounds **12**–**13**, **31**, and **32** activity was the same with moderate fungicidal effect (MIC = 250–500 µg/mL, MFC = 500–1000 µg/mL and MFC/MIC = 1–4). For compounds **2**, **6**, **12**, and **14**, the activity was weak or moderate (MIC = 500–1000 µg/mL, MFC > 1000 µg/mL) or not at all. The remaining substances did not show any antimycotic effects (Table 1 and Table 2). 

Detailed microbiological tables containing data on the activity of all compounds against all tested microbial strains can be found in the Supplementary Materials (Tables S1 and S2).

### 2.3. Cytotoxicity Studies

The 24 h and 48 h culture of L929 cells with 3-acetyl-2,5-disubstituted-1,3,4-oxadiazolines (**24**, **29**, **37**) showed that the tested compounds did not adversely affect the viability of normal cells. These compounds increased the viability of cells, especially during the 48 h culture (Table 3).

The 24 h and 48 h culture of A549 with compounds (**24**, **29**, **37**) showed that the best effect in promoting of the cytotoxicity was obtained using compound **37** at a concentration of 100 and 50 µM during the 24 h culture. Other compounds did not produce such effect, and sometimes quite the opposite—an increase of cell viability (Table 4).

The 24 h and 48 h culture of HeLa cells with (**24**, **29**, **37**) derivatives showed that the main effect was the increase of cell viability. There was no decrease in cell cytotoxicity (Table 5). All tested compounds belonged to the group of 3-acetyl-2,5-disubstituted-1,3,4-oxadiazolines and were derived from 3-methyl-4-nitrobenzhydrazide **1**. Compound **24** possessed 5-(4-chlorophenyl)furan-2-yl scaffold in second position of 1,3,4-oxadiazole ring whereas compound **29** had 4-(pyridin-2-yl)phenyl in the same position and compound **37** had quinolin-4-yl substituent in the second position of 1,3,4-oxadiazole ring.

### 2.4. SAR Analysis

Based on our microbiological studies, it can be concluded that the activity of 3-acetyl-1,3,4-oxadiazole derivatives is greater than that of the corresponding hydrazide–hydrazones. On this basis, it can be concluded that compounds containing two heterocyclic rings are more active than compounds containing only one. Ideal confirmation of this theory are compounds **2** and **20**. Compounds **2** and **20** had the same substituent 5-iodofuran but compound **2** was a hydrazide-hydrazone and compound **20** was a 1,3,4-oxadiazole derivative. The cyclization product had two times better activity against most of the tested bacterial strains.

Structure-activity analysis carried out on the 1,3,4-oxadiazole molecules showed that the position 2 and 5 are a critical site of modification of these kind of molecules and play a dominant role in defining the pharmacological activity of synthesized derivatives [11,14,17,46]. The most active compound of the series was **37** which had quinolin-4-yl substituent in second position of 1,3,4-oxadiazole ring and 3-methyl-4-nitrophenyl in position 5. The *Staphylococcus aureus* ATCC 25923 strain turned out to be the most sensitive to hydrazide—hydrazones. On the other hand, among Gram-positive, the most sensitive to oxadiazoles was *Staphylococcus epidermidis* ATCC 12228, among Gram-negative *Bordetella bronchiseptica* ATCC 4617, and among *Candida albicans* ATCC 2091 fungi. Compound **2** turned out to be the most active among compounds **2**–**19**, and causes a drastic decrease in activity in relation to all tested strains. In compound **11**, the pyridin-2-ylphenyl substituent appears to be a significant fragment in building activity against Gram-negative bacteria. The replacement of atom I (compound **2**) with a Cl atom (compound **18**) causes a very marked increase in activity in relation to *Candida* spp. Moreover, changing the substitution in the quinoline ring from 4 to 2 drastically lowers the activity. On the basis of compounds **13** and **14**, it can also be concluded that the change of the substituent ring from the six-membered to the five-membered causes a decrease in activity against *Micrococcus luteus* ATCC 10240 and *Bacillus subtilis* ATCC 6633.

The conversion of the two most active hydrazones into the appropriate oxadiazoles increases their activity against all strains tested. Replacing the iodine atom with a nitro or 4-chlorophenyl group causes a decrease in activity against the tested Gram-positive, Gram-negative and fungi. Conversely, replacing the iodine atom with chlorine causes a decrease in activity in relation to Gram-positive, Gram-negative, but an increase in relation to most *Candida* fungi. On the basis of compounds **31** and **32**, it can be concluded that replacing the 5-membered substituent with the 6-membered substituent causes a two-fold decrease in activity in relation to *C. albicans* ATCC 2091, however, it does not change the activity in relation to other *Candida* spp. 

Comparing the activity of hydrazones and oxadiazoles, it can be stated that hydrazones are a group of compounds showing higher activity in relation to Gram-positive bacteria. *S. aureus* ATCC 25923 turned out to be the most sensitive strain to hydrazones and *S. epidermidis* ATCC 12228 to oxadiazoles. Both groups slightly inhibit the multiplication of Gram-negative bacteria. In both cases, the most sensitive strain is *B. bronchiseptica* ATCC 4617. Analyzing the activity against fungi of the strains *Candida*, it can be concluded that oxadiazoles are characterized by higher activity in relation to all strains.

More detailed studies are reasonable to determine the additional physicochemical and biological parameters to get further insight into the relationship between structure and activity and optimize the performance of this series of molecules.

## 3. Material and Methods

### 3.1. Chemistry

All reagents used for the experiments were purchased from Sigma-Aldrich (Munich, Germany) and Merck Co. (Darmstadt, Germany) and used without further purification. They had a class of the purity declared by the manufacturer. The melting points of the obtained compounds were determined with a Fisher–Johns apparatus (Fisher Scientific, Schwerte, Germany), without any correction. The purity of the compounds obtained was assessed through thin layer chromatography (TLC) on plates covered with silica gel (aluminum oxide 60 F-254) by Merck. Chloroform-ethanol mixtures in the ratio 10:1 (*v*/*v*) were used as the mobile phase. Spots were developed by irradiation with UV light with a wavelength λ = 254 nm. FT-IR spectra were recorded on a Nicolet 6700 spectrometer (Thermo Scientific, USA); in cm^−1^. ^1^H and ^13^C NMR spectra were recorded on the Bruker Avance 300 and 600 apparatus (Bruker BioSpin GmbH, Hamburg, Germany). The compounds were dissolved in dimethyl sulfoxide (DMSO-*d*_6_) for analysis. Tetramethylsilane (TMS) was used as an internal standard. Chemical shift values are given in ppm. Then elemental analyses were determined by a Perkin Elmer 2400 series II CHNS/O analyzer (PerkinElmer, Waltham, MA, USA), and the results were within ±0.4% of the theoretical value.

### 3.2. Synthesis of Hydrazide–Hydrazones of 3-Methyl-4-nitrobenzoic Acid (***2***–***19***)

New hydrazide–hydrazones of 3-methyl-4-nitrobenzoic acid (**2**–**19**) were obtained on the basis of the procedure reported earlier [47,48]. The 3-Methyl-4-nitrobenzhydrazide (0.01 mole) was dissolved in 10 mL of ethanol (96%) and appropriately substituted aldehyde (0.01 mole) was added. The mixture was heated under reflux for 3 h. Subsequently, the solution was allowed to cool and placed in refrigerator for 24 h. The precipitate was filtered off and recrystallized from ethanol.

Physico–chemical properties of hydrazide–hydrazones of 3-methyl-4-nitrobenzoic acid (**2**–**19**) are presented in Supplementary Materials.

### 3.3. Synthesis of 3-Acetyl-2,5-disubstituted-1,3,4-oxadiazolines (***20***–***37***)

The 0.001 mole of appropriate hydrazide–hydrazone of 3-methyl-4-nitrobenzhydrazide (**2**–**19**) was dissolved in 3 mL of neat acetic anhydride and heated under reflux for 3 h. Subsequently, the acetic anhydride was removed under reduced pressure. The compound which remains in the flask was cooled to room temperature and crushed ice was added to the flask. After that, it was shaken vigorously for about 5 min and left at room temperature for 24 h. Then, the precipitate formed was filtered off and recrystallized from ethanol:acetone (3:1 *v*/*v*) mixture.

Physico–chemical properties of 3-acetyl-2,5-disubstituted-1,3,4-oxadiazolines (**20**–**37**) are presented in Supplementary Materials.

### 3.4. Microbiology

The in vitro screening of compounds **2**–**37** was performed with the use of the broth microdilution method as described earlier by our group [47,48] and according to the European Committee on Antimicrobial Susceptibility Testing (EUCAST) and Clinical and Laboratory Standards Institute guidelines [49,50]. We used a panel of reference and clinical or saprophytic strains of microorganisms from American Type Culture Collection (ATCC) in microbiology assays. All of the experiments were repeated three times, and representative data are presented. All of the stock solutions of the tested compounds were dissolved in DMSO.

### 3.5. Cytotoxicity Studies

#### 3.5.1. Cell Lines

The A549 cell line were obtained from ECACC and maintained in DMEM or EMEM, respectively, supplemented with 2 mM of glutamine, 10% fetal bovine serum, and antibiotics (1% of 100 U/L penicillin, 100 mg/mL streptomycin). L929 (NCTC clone 929, ATCC^®^ CCL-1™) and HeLa (ATCC^®^ CCL-2™) cell lines were obtained from ATCC and maintained in EMEM supplemented with antibiotics (1% of 100 U/L penicillin, 100 mg/mL streptomycin) and with 5% and 10% FBS respectively. Cell line was routinely grown in tissue culture flasks (75 cm^2^) and kept in a humidified atmosphere of 5% CO_2_ at 37 °C. All examined cell lines was tested against mycoplasma contamination with microbiological assays. The research was carried out using the normal cell line L929—mouse fibroblast and cancer cells: HeLa—human cervical cancer, A549—human lung cancer. All of the stock solutions of the tested compounds were dissolved in DMSO.

#### 3.5.2. MTT Analysis

To determine tested compounds cytotoxicity, an indirect method was used—using spectrophotometric measurement of product concentration resulting from the reduction of the chemical compound by living cells (with functional mitochondria). The amount of colored product is directly proportional to the metabolic activity of the cell.

The principle of the assay is based on the ability of live cells, with intact mitochondrial membrane, to reduce water-insoluble yellow 3-(4,5-dimethyl-1,3-thiazol-2-yl)-2,5-diphenyl-2*H*-tetrazole bromide—MTT) to purple formazan, also insoluble in water. Therefore, L929, A549, and HeLa cell lines were cultured for 24 h in 96-well plates in order to adhere well to the basis. Then 20 μL of tested compounds (range 6–200 μM) were added to the cells—compounds marked as **24**, **29**, **37** were tested on L929, A549, and HeLa cell lines. The untreated chosen cell lines were used as the control groups. After 24 and 48 h of incubation, 10 µL of MTT was added to the cells. Then, after 3 h, 100 µL of the medium was withdrawn from each well and quenched with the same amount of DMSO. After 5 min of incubation, absorbance at 570 nm was read on a BioTek model EPOCH ELISA plate reader. The absorbance values of the samples ranged from about 0.3 to about 1.2 and, therefore, were within the range of linearity of the Lambert–Beer law.

## 4. Conclusions

We synthesized nineteen novel hydrazide–hydrazones (**2**–**19**) and nineteen novel 2,5-distubstituted 3-acetyl-1,3,4-oxadiazole derivatives (**20**–**37**) and evaluated them for their in vitro antimicrobial activity and cytotoxicity.

Microbiological tests showed that the most active of the tested compounds (**2**–**37**) was compound **37**, which showed activity against all tested bacteria strains (except *K. pneumoniae*, *P. mirabilis*, and *P. aeruginosa*), and fungi. *S. epidermidis* was sensitive to all studied compounds. Moreover, 1,3,4-oxadiazole derivatives (**20**–**37**) showed strong or very strong bactericidal effect, especially against *Staphylococcus* spp. belonging to Gram-positive bacteria.

Furthermore, cytotoxicity studies showed that compounds marked as **24**, **29**, **37** turned out to be the best acting compounds (especially number **37**), and the line that was most sensitive to them was A549. During 24 h incubation, a decrease in viability of 58% (at dose 100 µM) and 56% (at dose 50 µM) was noted here, along with a high viability of normal cells. Compounds marked as **24**, **29**, **37** did not possess antiproliferative properties against checked tumor cell lines. On the contrary, after their use, an increase in proliferation was noticed. Of course, it does not mean that these compounds have carcinogenic properties. An increase in proliferation could mean that these compounds are neutral to cancer cells, and their divisions were the result of the natural cell cycle.

Furthermore, we observed significantly higher activity of compounds having a 3-acetyl-1,3,4-oxadiazole (**20**–**39**) ring compared to the initial hydrazide–hydrazones (**2**–**19**).

The compound with the most promising antibacterial and cytotoxicity profile was 3-acetyl-1,3,4-oxadiazole (**37**), with possessed quinolin-4-yl substituent in the second position of oxadiazole ring and 3-methyl-4-nitrophenyl in position 5. This compound showed strong or very strong activity especially against *S. epidermidis* (MIC = 0.48 µg/mL).

Based on the obtained antimicrobial activity and cytotoxicity results, it seems practical to still search for active molecules among this group of compounds in the future.

## Figures and Tables

**Table 1:** The best active compounds expressed as minimal inhibitory concentration (MIC) (minimal bactericidal concentration (MBC) or (MFC) (minimum fungicidal concentration) (µg/mL) and (MBC/MIC or MFC/MIC) values against the reference strains of microorganisms.

Species/Compound No.		2	4	11	13	14	15	18	19	CIP/NY *	NIT	CFX	APC
**Gram-positive bacteria**	*Staphylococcus aureus* ATCC 25923	31.25 (62.5) ****{2}****	250 (>1000) {>4}	500 (>1000) ****{>2}****	125 (>1000) {>8}	250 (>1000) {>4}	125 (>1000) {>8}	500 (1000) ****{2}****	500 (1000) ****{2}****	0.48 (0.48) {1}	15.62 (15.62)	0.49	nd
	*Staphylococcus aureus* ATCC 6538	62.5 (125) ****{2}****	500 (>1000) **{>2}**	500 (>1000) **{>2}**	250 (>1000) {>4}	250 (>1000) {>4}	1000 (>1000) **{>1}**	500 (1000) **{2}**	500 (1000) **{2}**	0.24 (0.24) {1}	15.62 (15.62)	0.98	nd
	*Staphylococcus aureus* ATCC 43300	125 (500) **{4}**	500 (>1000) **{>2}**	500 (>1000) **{>2}**	1000 (>1000) **{>1}**	1000 (>1000) **{>1}**	1000 (>1000) **{>1}**	500 (1000) **{2}**	500 (1000) **{2}**	0.24 (0.24) {1}	7.81 (15.62)	nd	nd
	*Staphylococcus aureus* ATCC 29213	125 (500) **{4}**	500 (>1000) **{>2}**	500 (>1000) **{>2}**	500 (>1000) **{>2}**	500 (>1000) **{>2}**	1000 (>1000) **{>1}**	500 (1000) **{2}**	500 (1000) **{2}**	0.48 (0.48) {1}	nd	nd	nd
	*Staphylococcus epidermidis* ATCC 12228	15.62 (15.62) **{1}**	250 (>1000) {>4}	250 (>1000) {>4}	500 (>1000) **{>2}**	250 (>1000) {>4}	500 (>1000) **{>2}**	500 (1000) **{2}**	500 (1000) **{2}**	0.12 (0.12) {1}	3.91 (7.81)	0.24	nd
	*Enterococcus faecalis* ATCC 29212	1000 (>1000) **{>1}**	1000 (>1000) **{>1}**	500 (>1000) **{>1}**	500 (>1000) **{>2}**	500 (>1000) **{>2}**	1000 (>1000) **{>1}**	500 (1000) **{2}**	500 (1000) **{2}**	0.98 (1.95) {2}	nd	nd	nd
	*Micrococcus luteus* ATCC 10240	1000 (>1000) **{>1}**	125 (>1000) {>8}	250 (>1000) {>4}	250 (>1000) {>4}	31.25 (>1000) {>32}	500 (>1000) **{>2}**	500 (1000) **{2}**	500 (1000) **{2}**	0.98 (1.95) {2}	62.5 (62.5)	0.98	nd
	*Bacillus subtilis* ATCC 6633	62.5 (125) **{2}**	500 (>1000) **{>2}**	125 (>1000) {>8}	250 (>1000) {>4}	31.25 (>1000) {>32}	125 (>1000) {>8}	500 (500) {1}	500 (500) {1}	0.03 (0.03) {1}	3.91 (3.91)	15.62	62.5
	*Bacillus cereus* ATCC 10876	1000 (>1000) **{>1}**	1000 (>1000) **{>1}**	500 (>1000) **{>2}**	500 (>1000) **{>2}**	1000 (>1000) **{>1}**	1000 (>1000) **{>1}**	500 (1000) **{2}**	500 (1000) **{2}**	0.06 (0.12) {2}	7.81 (15.62)	31.25	nd
**Gram-negative bacteria**	*Bordetella bronchiseptica* ATCC 4617	-	1000 (>1000) **{>1}**	500 (>1000) **{>2}**	-	-	-	500 (1000) **{2}**	500 (1000) **{2}**	0.98 (0.98) {1}	125 (>1000)	nd	nd
**Fungi**	*Candida albicans* ATCC 2091	1000 (>1000) **{>1}**	-	1000 (>1000) **{>1}**	-	-	-	125 (500) **{4}**	125 (500) **{4}**	0.24 * (0.24) **{1}**	na	na	na
	*Candida albicans* ATCC 10231	1000 (>1000) **{>1}**	500 (>1000) **{>2}**	500 (>1000) **{>2}**	-	-	-	62.5 (250) **{4}**	62.5 (250) **{4}**	0.48 * (0.48) **{1}**	na	na	na
	*Candida parapsilosis* ATCC 22019	1000 (>1000) **{>1}**	1000 (>1000) **{>1}**	500 (>1000) **{>2}**	1000 (>1000) **{>1}**	-	-	125 (250) **{2}**	125 (250) **{2}**	0.24 * (0.48) {2}	na	na	na

“-” no activity; nd, not determined; na, not applicable; the standard chemotherapeutics used as positive controls: ciprofloxacin (CIP), nitrofurantoin (NIT), cefuroxime (CFX) and ampicillin (APC) for bacteria and nystatin (NY *), Compounds with bactericidal effect (MBC/MIC ≤4) or fungicidal effect (MFC/MIC ≤4), MBC/MIC or MFC/MIC in these cases are bolded. No bioactivity–MIC > 1000 µg/mL; mild bioactivity–MIC = 501–1000 µg/mL; moderate bioactivity–MIC = 126–500 µg/mL; good bioactivity–MIC = 26–125 µg/mL; strong bioactivity–MIC = 10–25 µg/mL; very strong bioactivity–MIC < 10 µg/mL. * Nystatin activity, ATCC—American Type Culture Collection.

**Table 2:** The best active compounds **20**–**37** expressed as MIC (MBC or MFC) (µg/mL) and (MBC/MIC or MFC/MIC) values against the reference strains of microorganisms.

Species/Compound No		20	24	25	30	31	32	37	CIP/VA */NY **	NIT	CFX	APC
**Gram-positive bacteria**	*Staphylococcus aureus* ATCC 25923	15.62 (15.62) **{1}**	1000 (>2000) **{>2}**	-	1000 (>2000) **{>2}**	1000 (2000) **{2}**	500 (1000) **{2}**	7.81 (7.81) **{1}**	0.48 (0.48) {1}	15.62 (15.62)	0.49	nd
	*Staphylococcus aureus* ATCC 6538	15.62 (15.62) **{1}**	1000 (>2000) **{>2}**	125 (>2000) {>16}	-	1000 (2000) **{2}**	500 (2000) **{4}**	7.81 (7.81) **{1}**	0.24 (0.24) {1}	15.62 (15.62)	0.98	nd
	*Staphylococcus aureus* ATCC 43300	15.62 (15.62) **{1}**	1000 (>2000) **{>2}**	-	-	-	1000 (2000) **{2}**	15.62 (15.62) **{1}**	0.24 (0.24) {1}	7.81 (15.62)	nd	nd
	*Staphylococcus aureus* ATCC 29213	15.62 (15.62) **{1}**	500 (>2000) {>4}	-	1000 (>2000) **{>2}**	-	500 (500) **{1}**	15.62 (15.62) **{1}**	0.48 (0.48) {1}	Nd	nd	nd
	*Staphylococcus epidermidis* ATCC 12228	1.95 (3.91) **{2}**	250 (250) **{1}**	62.5 (125) **{2}**	7.81 (15.62) **{2}**	31.25 (125) **{4}**	62.5 (125) **{2}**	0.48 (0.48) **{1}**	0.12 (0.12) {1}	3.91 (7.81)	0.24	nd
	*Enterococcus faecalis* ATCC 29212	125 (500) **{4}**	500 (>2000) {>4}	-	1000 (>2000) **{>2}**	-	500 (>2000) {>4}	500 (1000) **{2}**	0.98* (1.95) {2}	Nd	nd	nd
	*Micrococcus luteus* ATCC 10240	125 (250) **{2}**	1000 (2000) **{2}**	250 (2000) {8}	1000 (>2000) **{>2}**	500 (1000) **{2}**	250 (500) **{2}**	62.5 (62.5) **{1}**	0.98 (1.95) {2}	62.5 (62.5)	0.98	nd
	*Bacillus subtilis* ATCC 6633	31.25 (62.5) **{2}**	125 (2000) {16}	-	1000 (2000) **{2}**	-	1000 (2000) **{2}**	31.25 (31.25) **{1}**	0.03 (0.03) {1}	3.91 (3.91)	15.62	62.5
	*Bacillus cereus* ATCC 10876	31.25 (31.25) **{1}**	125 (>2000) {>16}	-	1000 (>2000) **{>2}**	1000 (>2000) **{>2}**	500 (>2000) {>4}	31.25 (62.5) **{2}**	0.06 (0.12) {2}	7.81 (15.62)	31.25	nd
**Gram-negative bacteria**	*Bordetella bronchiseptica* ATCC 4617	125 (500) **{4}**	1000 (>2000) **{>2}**	-	1000 (2000) **{2}**	1000 (1000) **{1}**	1000 (2000) **{2}**	1000 (2000) **{2}**	0.98 (0.98) {1}	125 (>1000)	nd	nd
**Fungi**	*Candida albicans* ATCC 2091	31.25 (62.5) **{2}**	250 (2000) {8}	1000 (2000) **{2}**	1000 (2000) **{2}**	250 (500) **{2}**	500 (500) **{1}**	15.62 (15.62) **{1}**	0.24 ** (0.24) {1}	na	na	na
	*Candida parapsilosis* ATCC 22019	125 (500) **{4}**	62.5 (1000) {16}	1000 (2000) **{2}**	1000 (2000) **{2}**	500 (500) **{1}**	500 (500) **{1}**	250 (2000) {8}	0.24 ** (0.48) {2}	na	na	na

“-” no activity; nd, not determined; na, not applicable; the standard chemotherapeutics used as positive controls: ciprofloxacin (CIP), nitrofurantoin (NIT), cefuroxime (CFX), vancomycin (VA *), and ampicillin (APC) against bacteria and nystatin (NY **) for fungi. Compounds with bactericidal effect (MBC/MIC ≤ 4) or fungicidal effect (MFC/MIC ≤ 4), MBC/MIC or MFC/MIC in these cases are bolded. No bioactivity—MIC > 1000 µg/mL; mild bioactivity—MIC = 501–1000 µg/mL; moderate bioactivity—MIC = 126–500 µg/mL; good bioactivity—MIC = 26–125 µg/mL; strong bioactivity—MIC = 10–25 µg/mL; very strong bioactivity—MIC < 10 µg/mL. * vancomycin activity, ** Nystatin activity.

**Table 3:** The cell proliferation in % according to control after 24 h and after 48 h exposition on studied compounds in L929 cell line.

Dose/Compound	24		29		37	
	After 24 h	After 48 h	After 24 h	After 48 h	After 24 h	After 48 h
200 µM	97%	109%	88%	97%	81%	89%
150 µM	113%	120%	81%	105%	85%	84%
100 µM	99%	107%	96%	105%	99%	76%
50 µM	87%	100%	104%	117%	88%	64%
25 µM	97%	112%	109%	92%	78%	79%
12 µM	102%	108%	124%	143%	92%	83%
6 µM	98%	121%	117%	132%	81%	85%

Green—increase in the metabolic activity of the cell.

**Table 4:** The cell proliferation in % according to control after 24 h and after 48 h exposition on studied compounds in A549 cell line**.**

Dose/Compound	24		29		37	
	After 24 h	After 48 h	After 24 h	After 48 h	After 24 h	After 48 h
200 µM	76%	73%	94%	102%	65%	84%
150 µM	71%	79%	97%	94%	79%	92%
100 µM	75%	84%	96%	93%	58%	67%
50 µM	65%	97%	106%	112%	56%	73%
25 µM	71%	106%	101%	78%	73%	66%
12 µM	100%	111%	98%	97%	64%	71%
6 µM	91%	99%	104%	103%	93%	88%

Red–decrease in the metabolic activity of the cell, green—increase in the metabolic activity of the cell.

**Table 5:** The cell proliferation in % according to control after 24 h and after 48 h exposition on studied compounds in HeLa cell line.

Dose/Compound	24		29		37	
	After 24 h	After 48 h	After 24 h	After 48 h	After 24 h	After 48 h
200 µM	92%	107%	101%	98%	106%	104%
150 µM	86%	132%	97%	111%	96%	110%
100 µM	105%	121%	103%	118%	99%	98%
50 µM	117%	113%	94%	118%	91%	87%
25 µM	112%	106%	115%	124%	83%	93%
12 µM	124%	138%	128%	127%	108%	117%
6 µM	126%	147%	115%	136%	114%	120%

Green—increase in the metabolic activity of the cell.

## Supplementary Materials

The following are available online. Table S1: The activity data of compounds **2**–**19** expressed as MIC (MBC or MFC) (µg/mL) and {MBC/MIC or MFC/MIC} values against the reference strains of microorganisms, Table S2: The activity data of compounds **20**–**37** expressed as MIC (MBC or MFC) (µg/mL) and {MBC/MIC or MFC/MIC} values against the reference strains of microorganisms. Figure S1: ^1^H NMR spectrum of compound **2**, Figure S2: ^13^C NMR spectrum of compound **2**, Figure S3: ^1^H NMR spectrum of compound **18**, Figure S4. ^13^C NMR spectrum of compound **18**, Figure S5: ^1^H NMR spectrum of compound **23**, Figure S6: ^13^C NMR spectrum of compound **23**, Figure S7: 1H NMR spectrum of compound **33**, Figure S8. ^13^C NMR spectrum of compound **33**.
