# Supplementary material for: Novel 3-Acetyl-2,5-disubstituted-1,3,4-oxadiazolines: Synthesis and Biological Activity
Source: Molecules. 2020 Dec 10;25(24):5844. doi: 10.3390/molecules25245844 (PMC7763531; doi:10.3390/molecules25245844)
Supplement: Supplementary file 1 [file molecules-25-05844-s001.pdf]

Novel 3-acetyl-2,5-disubstituted-1,3,4-oxadiazolines: synthesis and biological activity

Kinga Paruch[a*], Łukasz Popiołek[a], Anna Biernasiuk[b], Anna Hordyjewska[c], Anna Malm[b], Monika Wujec[a]

[a] Chair and Department of Organic Chemistry, Faculty of Pharmacy, Medical University of Lublin, 4A Chodźki Street, 20-093 Lublin, Poland,
*e-mail: kinga.paruch@umlub.pl
[b] Chair and Department of Pharmaceutical Microbiology, Faculty of Pharmacy, Medical University of Lublin, 1 Chodźki Street, 20-093 Lublin, Poland
[c] Chair and Department of Medicinal Chemistry, Faculty of Medical Dentistry Medical University of Lublin, 4A Chodźki Street, 20-093 Lublin, Poland

SUPPLEMANTARY MATERIALS

Detailed physico-chemical properties of hydrazide-hydrazones of 3-methyl-4-nitrobenzoic acid (**2-19**)

N-[(5-iodofuran-2-yl)methylidene]-3-methyl-4-nitrobenzohydrazide (**2**)
Yellowish powder, Yield: 86%, M.p.: 218°C (EtOH), IR: 3138 (N-H), 3062 (CH, arom.), 2872 (CH, aliph.), 1634 (C=O), 1513 (C=N), 1241, 1094 (C-OC), 1026 (N-N); ^{1}H NMR (600 MHz, DMSO-*d*$_6$): 2.59 (s, 3H, CH_3), 7.34-7.35 (d, 1H, ArH, *J* = 6 Hz), 7.82-7.83 (d, 1H, ArH, *J* = 6 Hz), 7.95-7.96 (d, 1H, ArH, *J* = 6 Hz), 8.02 (s, 1H, ArH), 8.14-8.15 (d, 1H, ArH, *J* = 6 Hz), 8.41 (s, 1H, =CH), 12.44 (s, 1H, NH); ^{13}C NMR (150 MHz, DMSO-*d*$_6$): 19.75 (CH_3), 115.04, 116.37, 125.13, 127.12, 132.62, 133.43, 136.89 (7C_{ar}), 137.11 (=CH), 151.34, 151.85, 152.51 (3C_{ar}), 162.36 (C=O). Anal. calc. for $C_{14}H_{10}IN_3O_4$ (399.14) (%): C 39.12; H 2.53; N 10.53. Found: C 39.25; H 2.51; N 10.50.

3-methyl-4-nitro-*N*-[(5-nitrofuran-2-yl)methylidene]benzohydrazide (**3**)
Yellowish powder, Yield: 59%, M.p.: 248°C (EtOH), IR: 3135 (N-H), 3015 (CH, arom.), 2873 (CH, aliph.), 1647 (C=O), 1521 (C=N), 1205, 1101 (C-OC), 1057 (N-N); ^{1}H NMR (600 MHz, DMSO-*d*$_6$): 2.58 (s, 3H, CH_3), 6.90-6.91 (d, 1H, ArH, *J* = 6 Hz), 6.92-6.93 (d, 1H, ArH, *J* = 6 Hz), 7.91-7.93 (d, 1H, ArH, *J* = 12 Hz), 7.99 (s, 1H, ArH), 8.12-8.13 (d, 1H, ArH,

J = 6 Hz), 8.23 (s, 1H, =CH), 12.05 (s, 1H, NH); ^{13}C NMR (150 MHz, DMSO-d_6): 19.79 (CH_3), 97.20, 117.47, 122.87, 125.06, 126.95, 132.46, 133.36, 137.47 (8C_{ar}), 137.59 (=CH), 151.10, 154.48 (2C_{ar}), 161.95 (C=O). Anal. calc. for $C_{13}H_{10}N_4O_6$ (318.24) (%): C 49.06; H 3.17; N 17.61. Found: C 49.25; H 3.15; N 17.70.

N-[1*H*-indol-3-ylmethylidene]-3-methyl-4-nitrobenzohydrazide (**4**)

Yellow powder, Yield: 96%, M.p.: 212°C (EtOH), IR: 3400 (N-H), 3030 (CH, arom.), 2873 (CH, aliph.), 1607 (C=O), 1577 (C=N), 1205, 1101 (C-OC), 1053 (N-N); ^{1}H NMR (600 MHz, DMSO-d_6): 2.60 (s, 3H, CH_3), 7.16-7.19 (t, 1H, ArH, J = 12 Hz, J = 6 Hz), 7.21-7.24 (t, 1H, ArH, J = 6 Hz, J = 12 Hz), 7.45-7.47 (d, 1H, ArH, J = 12 Hz), 7.87-7.88 (d, 1H, ArH, J = 6 Hz), 7.94-7.96 (m, 1H, ArH), 8.02-8.03 (m, 1H, ArH), 8.12-8.13 (d, 1H, ArH, J = 6 Hz), 8.28-8.31 (t, 1H, ArH, J = 6 Hz, J = 12 Hz), 8.63 (s, 1H, =CH), 11.65 (s, 1H, NH), 11.73 (s, 1H, NH); ^{13}C NMR (150 MHz, DMSO-d_6): 19.90 (CH_3), 112.33. 112.87, 120.97, 121.27, 122.57, 123.91, 125.0, 126.86, 131.25, 132.27 (10C_{ar}), 137.53 (=CH), 138.90, 146.33, 150.80, 161.29 (4C_{ar}), 185.41 (C=O). Anal. calc. for $C_{17}H_{14}N_4O_3$ (322.32) (%): C 63.35; H 4.38; N 17.38. Found: C 63.5; H 4.37; N 17.30.

3-methyl-4-nitro-*N*-[quinolin-2-ylmethylidene]benzohydrazide (**5**)

Orange powder, Yield: 82%, M.p.: 118°C (EtOH), IR: 3426 (N-H), 3016 (CH, arom.), 2873 (CH, aliph.), 1655 (C=O), 1519 (C=N), 1229, 1092 (C-OC), 1057 (N-N); ^{1}H NMR (600 MHz, DMSO-d_6): 2.61 (s, 3H, CH_3), 7.65-7.68 (t, 1H, ArH, J = 12 Hz, J = 6 Hz), 7.80-7.83 (t, 1H, ArH, J = 12 Hz, J = 6 Hz), 7.98-7.99 (d, 2H, ArH, J = 6 Hz), 8.03-8.08 (m, 3H, ArH), 8.14-8.17 (m, 2H, ArH), 8.45-8.47 (d, 1H, ArH, J = 12 Hz), 8.62 (s, 1H, =CH), 12.42 (s, 1H, NH); ^{13}C NMR (150 MHz, DMSO-d_6): 19.79 (CH_3), 118.01, 125.12, 127.16, 127.94, 128.51, 129.43, 130.62, 132.59, 133.41, 134.04, 137.33 (11C_{ar}), 137.51 (=CH), 147.84, 149.34, 151.25, 154.02 (4C_{ar}), 162.44 (C=O). Anal. calc. for $C_{18}H_{14}N_4O_3$ (334.33) (%): C 64.66; H 4.22; N 16.76. Found: C 65.02; H 4.20; N 16.80.

N-{[5-(4-chlorophenyl)furan-2-yl]methylidene}-3-methyl-4-nitrobenzohydrazide (**6**)

Yellow powder, Yield: 99%, M.p.: 236°C (EtOH), IR: 3233 (N-H), 3015 (CH, arom.), 2873 (CH, aliph.), 1655 (C=O), 1522 (C=N), 1206, 1094 (C-OC), 1057 (N-N); ^{1}H NMR (600 MHz, DMSO-d_6): 2.60 (s, 3H, CH_3), 7.13-7.14 (d, 1H, ArH, J = 6 Hz), 7.23-7.24 (d, 1H, ArH, J = 6 Hz), 7.55-7.56 (d, 1H, ArH, J = 6 Hz), 7.83-7.85 (d, 1H, ArH, J = 12 Hz), 7.93-7.95 (d, 1H, ArH, J = 12 Hz), 8.01-8.02 (m, 1H, ArH), 8.13-8.14 (d, 1H, ArH, J = 6 Hz), 8.38 (s, 1H,

=CH), 12.04 (s, 1H, NH); ^{13}C NMR (150 MHz, DMSO-*d₆*): 19.81 (CH_3), 109.61, 117.26, 125.08, 126.20, 126.96, 128.76, 129.61, 132.44, 133.25, 133.37 ($12C_{ar}$), 137.72 (=CH), 138.51, 149.61, 151.09, 154.33 ($4C_{ar}$), 161.92 (C=O). Anal. calc. for $C_{19}H_{14}ClN_3O_4$ (383.79) (%): C 59.46; H 3.68; N 10.95. Found: C 60.15; H 3.65; N 10.90.

N-{[4-(1*H*-imidazol-1-yl)phenyl]methylidene}-3-methyl-4-nitrobenzohydrazide (**7**)

Yellow powder, Yield: 99%, M.p.: 192°C (EtOH), IR: 3626 (N-H), 2970 (CH, arom.), 2873 (CH, aliph.), 1683 (C=O), 1522 (C=N), 1204, 1101 (C-OC), 1059 (N-N); ^{1}H NMR (600 MHz, DMSO-d_6): 2.60 (s, 3H, CH_3), 7.15 (s, 1H, ArH), 7.79-7.81 (d, 2H, ArH, J = 12 Hz), 7.86 (s, 1H, ArH), 7.89-7.90 (d, 2H, ArH, J = 12 Hz), 7.95-7.96 (m, 1H, ArH), 8.02 (s, 1H, ArH), 8.13-8.14 (d, 1H, ArH, J = 6 Hz), 8.38 (s, 1H, ArH), 8.50 (s, 1H, =CH), 12.14 (s, 1H, NH); ^{13}C NMR (150 MHz, DMSO-d_6): 19.82 (CH_3), 118.27, 120.86, 125.05, 127.01, 129.15, 130.62, 132.48, 132.92, 133.33, 136.02 ($12C_{ar}$), 137.73 (=CH), 138.53, 148.11, 151.09 ($3C_{ar}$), 161.92 (C=O). Anal. calc. for $C_{18}H_{15}N_5O_3$ (349.34) (%): C 61.89; H 4.33; N 20.05. Found: C 62.15; H 4.35; N 20.15.

N-{[5-(3-chloro-4-methoxyphenyl)furan-2-yl]methylidene}-3-methyl-4-nitrobenzohydrazide (**8**)

Orange powder, Yield: 98%, M.p.: 182°C (EtOH), IR: 3271 (N-H), 3016(CH, arom.), 2873 (CH, aliph.), 1665 (C=O), 1519 (C=N), 1217, 1101 (C-OC), 1061 (N-N); ^{1}H NMR (600 MHz, DMSO-d_6): 2.59 (s, 3H, CH_3), 3.92 (s, 3H, CH_3, 7.10-7.11 (d, 1H, ArH, J = 6 Hz), 7.15-7.16 (d, 1H, ArH, J = 6 Hz), 7.27-7.28 (d, 1H, ArH, J = 6 Hz), 7.75-7.77 (m, 1H, ArH), 7.87-7.88 (d, 1H, ArH, J = 6 Hz), 7.93-7.95 (m, 1H, ArH), 8.00-8.01 (m, 1H, ArH), 8.13-8.14 (d, 1H, ArH, J = 6 Hz), 8.38 (s, 1H, =CH), 12.04 (s, 1H, NH); ^{13}C NMR (150 MHz, DMSO-d_6): 19.91 (CH_3), 56.79 (OCH_3), 108.39, 113.84, 117.49, 122.30, 123.56, 124.64, 125.08, 126.95, 132.42, 133.37, 137.74 ($11C_{ar}$), 138,61 (=CH), 149.04, 154.24, 155.01, 161.88 ($4C_{ar}$), 178.06 (C=O). Anal. calc. for $C_{20}H_{16}ClN_3O_5$ (413.81) (%): C 58.05; H 3.90; N 10.15. Found: C 59.15; H 3.85; N 10.20.

N-[(1*H*-imidazol-4-ylmethylidene]-3-methyl-4-nitrobenzohydrazide (**9**)

Yellow powder, Yield: 77%, M.p.: 310°C (EtOH), IR: 3726 (N-H), 3018 (CH, arom.), 2874 (CH, aliph.), 1690 (C=O), 1514 (C=N), 1217, 1092 (C-OC), 1045 (N-N); ^{1}H NMR (600 MHz, DMSO-d_6): 2.61 (s, 3H, CH_3), 7.58 (s, 1H, ArH), 7.78-7.80 (d, 1H, ArH, J = 12 Hz), 7.91-7.95 (m, 1H, ArH), 7.99-8.01 (d, 1H, ArH, J = 12 Hz), 8.11-8.17 (m, 1H, ArH), 8.38 (s, 1H,

=CH), 12.91 (s, 1H, NH), 14.48 (s, 1H, NH); ^{13}C NMR (150 MHz, DMSO-d_6): 19.95 (CH_3), 122.45, 125.54, 126.14, 126.90, 132.08, 133.83, 136.04, 137.21 ($8C_{ar}$), 137.91 (=CH), 151.15 (C_{ar}), 161.18 (C=O). Anal. calc. for $C_{12}H_{11}N_5O_3$ (273.25) (%): C 52.75; H 4.06; N 25.63. Found: C 52.65; H 4.00; N 25.59.

3-methyl-*N*-{[4-(morpholin-4-yl)phenyl]methylidene}-4-nitrobenzohydrazide (**10**)
Yellow crystals, Yield: 92%, M.p.: 198°C (EtOH), IR: 3726 (N-H), 3026 (CH, arom.), 2870 (CH, aliph.), 1651 (C=O), 1514 (C=N), 1227, 1062 (C-OC), 1048 (N-N); ^{1}H NMR (600 MHz, DMSO-d_6): 2.59 (s, 3H, CH_3), 3.22-3.24 (m, 4H, $2xCH_{2\text{-morpholine}}$), 3.74-3.76 (m, 4H, $2xCH_{2\text{-morpholine}}$), 7.02-7.03 (d, 2H, ArH, *J* = 6 Hz), 7.60-7.62 (d, 2H, ArH, *J* = 12 Hz), 7.92-7.93 (d, 1H, ArH, *J* = 6 Hz), 7.99 (s, 1H, ArH), 8.11-8.12 (d, 1H, ArH, *J* = 6 Hz), 8.34 (s, 1H, =CH), 11.85 (s, 1H, NH); ^{13}C NMR (150 MHz, DMSO-d_6): 19.84 (CH_3), 47.86 ($2xCH_{2\text{-morpholine}}$), 66.41 ($2xCH_{2\text{-morpholine}}$), 114.79, 124.64, 125.0, 126.89, 128.93, 132.36, 133.28 ($9C_{ar}$), 138.08 (=CH), 149.48, 150.93, 152.83 ($3C_{ar}$), 161.61 (C=O). Anal. calc. for $C_{19}H_{20}N_4O_4$ (368.39) (%): C 61.95; H 5.47; N 15.21. Found: C 61.25; H 5.50; N 15.32.

3-methyl-4-nitro-*N*-{[4-(pyridin-2-yl)phenyl]methylidene}benzohydrazide (**11**)
Yellow powder, Yield: 96%, M.p.: 220°C (EtOH), IR: 3455 (N-H), 3055 (CH, arom.), 2929 (CH, aliph.), 1651 (C=O), 1515 (C=N), 1258, 1067 (C-OC), 1011 (N-N); ^{1}H NMR (600 MHz, DMSO-d_6): 2.60 (s, 3H, CH_3), 7.39-7.41 (m, 1H, ArH), 7.88-7.89 (d, 2H, ArH, *J* = 6 Hz), 7.91-7.94 (m, 1H, ArH), 7.96-7.97 (d, 1H, ArH, *J* = 6 Hz), 8.03-8.06 (m, 2H, ArH), 8.13-8.14 (d, 1H, ArH, *J* = 6 Hz), 8.21-8.23 (d, 2H, ArH, *J* = 12 Hz), 8.52 (s, 1H, =CH), 8.70-8.71 (d, 1H, ArH, *J* = 6 Hz), 12.14 (s, 1H, NH); ^{13}C NMR (150 MHz, DMSO-d_6): 19.82 (CH_3), 120.95, 123.46, 125.05, 127.03, 127.39, 128.11, 132.49, 133.33, 135.09 ($11C_{ar}$), 137.80 (=CH), 140.65, 144.53, 148.61, 150.13, 151.09, 155.59 ($6C_{ar}$), 162.04 (C=O). Anal. calc. for $C_{20}H_{16}N_4O_3$ (360.37) (%): C 66.66; H 4.48; N 15.55. Found: C 67.15; H 4.51; N 15.52.

3-methyl-4-nitro-*N*-[(1*H*-pyrrol-2-ylmethylidene]benzohydrazide (**12**)
Yellow powder, Yield: 75%, M.p.: 204°C (EtOH), IR: 3436 (N-H), 3016 (CH, arom.), 2970 (CH, aliph.), 1648 (C=O), 1517 (C=N), 1217, 1097 (C-OC), 1027 (N-N); ^{1}H NMR (600 MHz, DMSO-d_6): 2.59 (s, 3H, CH_3), 6.16-6.17 (m, 1H, ArH), 6.53-6.54 (m, 1H, ArH), 6.94-6.95 (m, 1H, ArH), 7.92-7.93 (d, 1H, ArH, *J* = 6 Hz), 8.00-8.01 (m, 1H, ArH), 8.10-8.12 (d, 1H, ArH, *J* = 12 Hz), 8.29 (s, 1H, =CH), 11.60 (s, 1H, NH), 11.75 (s, 1H, NH); ^{13}C NMR (150 MHz, DMSO-d_6): 19.83 (CH_3), 109.95, 114.32, 123.55, 124.99, 126.84, 127.27, 132.34,

133.29 (8C_{ar}), 138.16 (=CH), 142.15, 150.88 (2C_{ar}), 161.40 (C=O). Anal. calc. for $C_{13}H_{12}N_4O_3$ (272.26) (%): C 57.35; H 4.44; N 20.58. Found: C 57.15; H 4.50; N 20.42.

3-methyl-4-nitro-*N*-{[4-(pyrrolidin-1-yl)phenyl]methylidene}benzohydrazide (**13**)
Orange powder, Yield: 66%, M.p.: 210°C (EtOH), IR: 3625 (N-H), 3015 (CH, arom.), 2830 (CH, aliph.), 1652 (C=O), 1548 (C=N), 1217, 1157 (C-OC), 1060 (N-N); ^{1}H NMR (600 MHz, DMSO-d_6): 1.97-1.99 (m, 4H, 2x$CH_{2\text{-pyrrolidine}}$), 2.59 (s, 3H, CH_3), 3.29-3.31 (m, 4H, 2x$CH_{2\text{-pyrrolidine}}$), 6.60-6.61 (d, 2H, ArH, J = 6 Hz), 7.54-7.56 (d, 2H, ArH, J = 12 Hz), 7.91-7.93 (d, 1H, ArH, J = 12 Hz), 7.99 (s, 1H, ArH), 8.10-8.12 (d, 1H, ArH, J = 12 Hz), 8.30 (s, 1H, =CH), 11.73 (s, 1H, NH); ^{13}C NMR (150 MHz, DMSO-d_6): 19.85 (CH_3), 25.38 (2x$CH_{2\text{-pyrrolidine}}$), 47.71 (2x$CH_{2\text{-pyrrolidine}}$), 111.74, 112.06, 121.11, 124.71, 124.99, 126.83, 129.23, 132.30 (10C_{ar}), 138.25 (=CH), 150.25, 152.13 (2C_{ar}), 190.12 (C=O). Anal. calc. for $C_{19}H_{20}N_4O_3$ (352.39) (%): C 64.76; H 5.72; N 15.90. Found: C 64.70; H 5.71; N 15.93.

3-methyl-4-nitro-*N*-{[4-(piperidin-1-yl)phenyl]methylidene}benzohydrazide (**14**)
Orange powder, Yield: 60%, M.p.: 172°C (EtOH), IR: 3458 (N-H), 3016 (CH, arom.), 2857 (CH, aliph.), 1654 (C=O), 1513 (C=N), 1229, 1067 (C-OC), 1026 (N-N); ^{1}H NMR (600 MHz, DMSO-d_6): 1.57-1.61 (m, 8H, 4x$CH_{2\text{-piperidine}}$), 2.54 (s, 3H, CH_3), 3.28-3.30 (m, 4H, 2x$CH_{2\text{-piperidine}}$), 6.98-7.00 (d, 2H, ArH, J = 12 Hz), 7.56-7.58 (d, 2H, ArH, J = 12 Hz), 7.92-7.93 (m, 1H, ArH), 7.99-8.00 (m, 1H, ArH), 8.04-8.05 (d, 1H, ArH, J = 6 Hz), 8.10-8.12 (d, 1H, ArH, J = 12 Hz), 8.32 (s, 1H, =CH), 11.80 (s, 1H, NH); ^{13}C NMR (150 MHz, DMSO-d_6): 19.84 (CH_3), 25.44 ($CH_{2\text{-piperidine}}$), 48.08 (2x$CH_{2\text{-piperidine}}$), 48.80 (2x$CH_{2\text{-piperidine}}$), 113.50, 115.0, 125.90, 126.88, 132.09, 133.27 (8C_{ar}), 138.13 (=CH), 149.64, 150.90, 152.98, 155.09 (4C_{ar}), 161.52 (C=O). Anal. calc. for $C_{20}H_{22}N_4O_3$ (366.41) (%): C 65.56; H 6.05; N 15.29. Found: C 65.15; H 6.51; N 15.36.

3-methyl-4-nitro-*N*-[pyridin-2-ylmethylidene]benzohydrazide (**15**)
White powder, Yield: 83%, M.p.: 140°C (EtOH), IR: 3551 (N-H), 3016 (CH, arom.), 2970 (CH, aliph.), 1669 (C=O), 1515 (C=N), 1217, 1103 (C-OC), 1050 (N-N); ^{1}H NMR (600 MHz, DMSO-d_6): 2.60 (s, 3H, CH_3), 7.45-7.47 (m, 1H, ArH), 7.90-7.93 (m, 1H, ArH), 7.96-7.97 (d, 1H, ArH, J = 6 Hz), 8.00-8.04 (m, 2H, ArH), 8.13-8.15 (d, 1H, ArH, J = 12 Hz), 8.49 (s, 1H, =CH), 8.64-8.65 (d, 1H, ArH, J = 6 Hz), 12.26 (s, 1H, NH); ^{13}C NMR (150 MHz, DMSO-d_6): 19.77 (CH_3), 120.54, 125.09, 127.1, 132.54, 133.37, 137.43 (6C_{ar}), 137.52 (=CH), 149.05,

149.91, 150.07, 151.21, 153.47 (5C_{ar}), 162.26 (C=O). Anal. calc. for $C_{14}H_{12}N_4O_3$ (284.27) (%): C 59.15; H 4.25; N 19.71. Found: C 59.15; H 4.21; N 19.42.

3-methyl-4-nitro-*N*-[pyridin-3-ylmethylidene]benzohydrazide (**16**)

Fluffy white powder, Yield: 79%, M.p.: 230°C (EtOH), IR: 3181 (N-H), 3016 (CH, arom.), 2970 (CH, aliph.), 1655 (C=O), 1513 (C=N), 1217, 1063 (C-OC), 1021 (N-N); ^{1}H NMR (600 MHz, DMSO-d_6): 2.59 (s, 3H, CH_3), 7.51-7.53 (m, 1H, ArH), 7.95-7.96 (d, 1H, ArH, *J* = 6 Hz), 8.02 (s, 1H, ArH), 8.13-8.14 (d, 1H, ArH, *J* = 6 Hz), 8.17-8.19 (d, 1H, ArH, *J* = 12 Hz), 8.52 (s, 1H, =CH), 8.64-8.65 (m, 1H, ArH), 8.89-8.90 (d, 1H, ArH, *J* = 6 Hz), 12.28 (s, 1H, NH); ^{13}C NMR (150 MHz, DMSO-d_6): 19.79 (CH_3), 124.53, 125.06, 127.04, 130.47, 132.51, 133.33, 134.06 (7C_{ar}), 137.57 (=CH), 146.49, 149.35, 151.16, 151.42 (4C_{ar}), 162.13 (C=O). Anal. calc. for $C_{14}H_{12}N_4O_3$ (284.27) (%):C 59.15; H 4.25; N 19.71.Found: C 59.35; H 4.25; N 19.62.

3-methyl-4-nitro-*N*-[thiophen-2-ylmethylidene]benzohydrazide (**17**)

Yellow crystals, Yield: 64%, M.p.: 226°C (EtOH), IR: 3166 (N-H), 3023 (CH, arom.), 2970 (CH, aliph.), 1652 (C=O), 1509 (C=N), 1217, 1060 (C-OC), 1040 (N-N); ^{1}H NMR (600 MHz, DMSO-d_6): 2.59 (s, 3H, CH_3), 7.16-7.18 (m, 1H, ArH), 7.52-7.53 (d, 1H, ArH, *J* = 6 Hz), 7.72-7.73 (d, 1H, ArH, *J* = 6 Hz), 7.91-7.93 (d, 1H, ArH, *J* = 12 Hz), 7.99 (s, 1H, ArH), 8.12-8.13 (d, 1H, ArH, *J* = 6 Hz), 8.66 (s, 1H, =CH), 12.02 (s, 1H, NH); ^{13}C NMR (150 MHz, DMSO-d_6): 19.81 (CH_3), 125.05, 126.94, 128.41, 129.84, 131.92, 132.40, 133.33 (7C_{ar}), 137.76 (=CH), 139.27, 144.24, 151.06 (3C_{ar}), 161.83 (C=O). Anal. calc. for $C_{13}H_{11}N_3O_3S$ (289.31) (%): C 53.97; H 3.83; N 14.52. Found: C 59.05; H 3.80; N 14.29.

N-[(5-chlorofuran-2-yl)methylidene]-3-methyl-4-nitrobenzohydrazide (**18**)

Fluffy white powder, Yield: 68%, M.p.: 218°C (EtOH), IR: 3137 (N-H), 3019 (CH, arom.), 2872 (CH, aliph.), 1661 (C=O), 1515 (C=N), 1222, 1085 (C-OC), 1027 (N-N); ^{1}H NMR (600 MHz, DMSO-d_6): 2.58 (s, 3H, CH_3), 6.71-6.72 (d, 1H, ArH, *J* = 6 Hz), 7.09-7.10 (d, 1H, ArH, *J* = 6 Hz), 7.91-7.93 (d, 1H, ArH, *J* = 12 Hz), 7.99 (s, 1H, ArH), 8.12-8.13 (d, 1H, ArH, *J* = 6 Hz), 8.25 (s, 1H, =CH), 12.08 (s, 1H, NH); ^{13}C NMR (150 MHz, DMSO-d_6): 19.78 (CH_3), 110.03, 117.10, 125.06, 126.95, 132.47, 133.36, 137.55 (7C_{ar}), 137.62 (=CH), 138.14, 149.44, 151.13 (3C_{ar}), 161.98 (C=O). Anal. calc. for $C_{13}H_{10}ClN_3O_4$ (307.69) (%): C 50.75; H 3.28; N 13.66. Found: C 51.98; H 3.25; N 13.52.

3-methyl-4-nitro-*N*-[quinolin-4-ylmethylidene]benzohydrazide (**19**)

Grey powder, Yield: 75%, M.p.: 206°C (EtOH), IR: 3236 (N-H), 3016 (CH, arom.), (2970 CH, aliph.), 1677 (C=O), 1515 (C=N), 1229, 1098 (C-OC), 1031 (N-N); ^{1}H NMR (600 MHz, DMSO-d_6): 2.62 (s, 3H, CH_3), 7.77-7.79 (t, 1H, ArH, J = 12 Hz), 7.85-7.88 (m, 2H, ArH), 8.01-8.02 (d, 1H, ArH, J = 6 Hz), 8.08 (s, 1H, ArH), 8.13-8.14 (d, 1H, ArH, J = 6 Hz), 8.17-8.18 (d, 1H, ArH, J = 6 Hz), 8.78-8.79 (d, 1H, ArH, J = 6 Hz), 9.02-9.03 (d, 1H, ArH, J = 6 Hz), 9.11 (s, 1H, =CH), 12.42 (s, 1H, NH); ^{13}C NMR (150 MHz, DMSO-d_6): 19.83 (CH_3), 120.66, 123.73, 124.66, 126.25, 128.15, 129.53, 130.04, 130.50, 132.55, 133.40, 136.86, 137.34 (12C_{ar}), 137.53 (=CH), 146.44, 148.92, 151.57 (3C_{ar}), 162.25 (C=O). Anal. calc. for $C_{18}H_{14}N_4O_3$ (334.33) (%): C 64.66; H 4.22; N 16.76. Found: C 64.15; H 4.31; N 16.82.

Physico-chemical properties of 3-acetyl-2,5-disubstituted-1,3,4-oxadiazolines (**20-37**).

1-[2-(5-iodofuran-2-yl)-5-(3-methyl-4-nitrophenyl)-1,3,4-oxadiazol-3(2*H*)-yl]ethanone (**20**)

Grey powder, Yield: 25%, M.p.: 120°C (EtOH:acetone 3:1), IR: 3053 (CH, arom.), 2840 (CH, aliph.), 1686 (C=O), 1585 (C=N), 1297, 1019 (C-OC); ^{1}H NMR (600 MHz, DMSO-d_6): 2.32 (s, 3H, CH_3), 2.58 (s, 3H, CH_3), 7.28-7.29 (d, 1H, ArH, J = 6 Hz), 7.44 (s, 1H, $CH_{oxadiazole}$), 7.73-7.74 (d, 1H, ArH, J = 6 Hz), 7.89-7.96 (m, 2H, ArH), 8.12-8.14 (d, 1H, ArH, J = 12 Hz); ^{13}C NMR (75 MHz, DMSO-d_6): 19.53 (CH_3), 21.58 (CH_3), 94.10 ($CH_{oxadiazole}$), 120.49, 125.08, 128.55, 129.64, 129.67, 130.11, 131.49, 133.30, 133.97, 135.01 (10C_{ar}), 151.96 ($C_{oxadiazole}$)), 166.33 (C=O). Anal. calc. for $C_{15}H_{12}IN_3O_5$ (441.18) (%): C 40.84; H 2.74; N 9.52 Found: C 41.45; H 2.68; N 9.52.

1-[5-(3-methyl-4-nitrophenyl)-2-(5-nitrofuran-2-yl)-1,3,4-oxadiazol-3(2*H*)-yl]ethanone (**21**)

Dark brown powder, Yield: 15%, M.p.: 148°C (EtOH:acetone 3:1), IR: 3078 (CH, arom.), 2860 (CH, aliph.), 1685 (C=O), 1585 (C=N), 1267, 1014 (C-OC); ^{1}H NMR (600 MHz, DMSO-d_6): 2.35 (s, 3H, CH_3), 2.55 (s, 3H, CH_3), 7.41 (s, 1H, $CH_{oxadiazole}$), 7.33-7.34 (d, 1H, ArH, J = 3 Hz), 7.70-7.71 (d, 1H, ArH, J = 6 Hz), 7.95-7.97 (d, 1H, ArH, J = 12 Hz), 8.04-8.07 (m, 2H, ArH); ^{13}C NMR (150 MHz, DMSO-d_6): 19.53 (CH_3), 23.17 (CH_3), 94.10 ($CH_{oxadiazole}$), 113.39, 114.75, 120.49, 125.06, 128.54, 131.49, 133.28, 133.96, 134.99, 146.55 (10C_{ar}), 151.97 ($C_{oxadiazole}$)), 166.31 (C=O). Anal. calc. for $C_{15}H_{12}N_4O_7$ (360.28) (%): C 50.01; H 3.36; N 15.55. Found: C 50.15; H 3.40; N 15.52.

1-[2-(1*H*-indol-3-yl)-5-(3-methyl-4-nitrophenyl)-1,3,4-oxadiazol-3(2*H*)-yl]ethanone (**22**)

Light brown powder, Yield: 12%, M.p.: 132°C (EtOH:acetone 3:1), IR: 3286 (N-H), 3031 (CH, arom.), 2970 (CH, aliph.), 1737 (C=O), 1519 (C=N), 1228, 1010 (C-OC); ^{1}H NMR (600 MHz, DMSO-d_6): 1.94 (s, 3H, CH_3), 2.77 (s, 3H, CH_3), 7.21-7.27 (m, 1H, ArH), 7.37 (s, 1H, $CH_{oxadiazole}$), 7.40-7.47 (m, 3H, ArH), 7.96 (s, 1H, ArH), 8.08-8.10 (d, 1H, ArH, J = 12 Hz), 8.15-8.17 (d, 1H, ArH, J = 12 Hz), 8.37-8.38 (d, 1H, ArH, J = 6 Hz), 10.09 (s, 1H, NH); ^{13}C NMR (DMSO-d_6): 19.55 (CH_3), 24.27 (CH_3), 88.41 ($CH_{oxadiazole}$), 116.57, 121.52, 121.63, 125.04, 125.44, 126.07, 126.66, 126.75, 128.51, 132.20, 136.21, 140.15 (12C_{ar}), 151.14 ($C_{oxadiazole)}$), 164.37, 168.88 (2C_{ar}) 170.72 (C=O). Anal. calc. for $C_{19}H_{16}N_4O_4$ (364.35) (%): C 62.63; H 4.43; N 15.38. Found: C 59.85; H 4.51; N 15.42.

1-[5-(3-methyl-4-nitrophenyl)-2-(quinolin-2-yl)-1,3,4-oxadiazol-3(2*H*)-yl]ethanone (**23**)
Dark brown powder, Yield: 26%, M.p.: 84°C (EtOH:acetone 3:1), IR: 3050 (CH, arom.), 2317 (CH, aliph.), 1669 (C=O), 1516 (C=N), 1260, 1036 (C-OC); ^{1}H NMR (600 MHz, DMSO-d_6): 2.33 (s, 3H, CH_3), 2.56 (s, 3H, CH_3), 7.36 (s, 1H, $CH_{oxadiazole}$), 7.68-7.71 (m, 1H, ArH), 7.73-7.75 (d, 1H, ArH, J = 12 Hz), 7.81-7.84 (m, 1H, ArH), 7.90-7.95 (m, 2H, ArH), 8.04-8.07 (m, 2H, ArH), 8.11-8.12 (d, 1H, ArH, J = 6 Hz), 8.51-8.53 (d, 1H, ArH, J = 12 Hz); ^{13}C NMR (75 MHz, DMSO-d_6): 19.95 (CH_3), 21.85 (CH_3), 93.81 ($CH_{oxadiazole}$), 120.00, 125.34, 126.06, 126.13, 128.37, 128.78, 128.84, 129.78, 131.12, 131.27, 134.64, 138.75, 147.73, 151.14, 154.05 (15C_{ar}), 155.16 ($C_{oxadiazole)}$), 167.59 (C=O). Anal. calc. for $C_{20}H_{16}N_4O_4$ (376.36) (%): C 63.82; H 4.28; N 14.28. Found: C 65.65; H 4.29; N 14.36.

1-{2-[5-(4-chlorophenyl)furan-2-yl]-5-(3-methyl-4-nitrophenyl)-1,3,4-oxadiazol-3(2*H*)-yl}ethanone (**24**)
Yellow powder, Yield: 17%, M.p.: 112°C (EtOH:acetone 3:1), IR: 3016 (CH, arom.), 2970 (CH, aliph.), 1661 (C=O), 1520 (C=N), 1290, 1040 (C-OC); ^{1}H NMR (600 MHz, DMSO-d_6): 2.39 (s, 3H, CH_3), 2.62 (s, 3H, CH_3), 7.36-7.37 (d, 1H, ArH, J = 6 Hz), 7.58-7.60 (d, 1H, ArH, J = 12 Hz), 7.59-7.61 (d, 2H, ArH, J = 12 Hz), 7.67-7.68 (d, 1H, ArH, J = 6 Hz), 7.89-7.61 (d, 1H, ArH, J = 12 Hz), 7.90-7.92 (d, 2H, ArH, J = 12 Hz), 8.36-8.38 (m, 1H, ArH), 9.63 (s, 1H, $CH_{oxadiazole}$); ^{13}C NMR (150 MHz, DMSO-d_6): 19.52 (CH_3), 21.05 (CH_3), 94.11 ($CH_{oxadiazole}$), 109.85, 125.04, 125.73, 126.19, 127.18, 127.96, 128.53, 129.76, 133.26, 133.95, 134.71, 135.01 (14C_{ar}), 151.94 ($C_{oxadiazole)}$), 152.31, 157.41 (2C_{ar}), 166.30 (C=O). Anal. calc. for $C_{21}H_{16}ClN_3O_5$ (425.82) (%): C 59.23; H 3.79; N 9.87. Found: C 58.65; H 3.79; N 9.80.

1-{2-[4-(1*H*-imidazol-1-yl)phenyl]-5-(3-methyl-4-nitrophenyl)-1,3,4-oxadiazol-3(2*H*)-yl}ethanone (**25**)

Yellow powder, Yield: 38%, M.p.: 80°C (EtOH:acetone 3:1), IR: 3012 (CH, arom.), 2970 (CH, aliph.), 1667 (C=O), 1515 (C=N), 1260, 1056 (C-OC); ^{1}H NMR (600 MHz, DMSO-d_6): 2.31 (s, 3H, CH_3), 2.57 (s, 3H, CH_3), 7.13-7.16 (m, 1H, ArH), 7.30 (s, 1H, $CH_{oxadiazole}$), 7.64-7.65 (d, 2H, ArH, J = 6 Hz), 7.73-7.74 (d, 2H, ArH, J = 6 Hz), 7.89-7.91 (d, 1H, ArH, J = 12 Hz), 7.95-7.96 (m, 1H, Ar), 8.04-8.06 (m, 1H, ArH), 8.11-8.12 (d, 1H, ArH, J = 6 Hz), 8.29-8.33 (m, 1H, ArH); ^{13}C NMR (75 MHz, DMSO-d_6): 19.70 (CH_3), 21.72 (CH_3), 92.60 ($CH_{oxadiazole}$), 121.19, 125.08, 125.79, 125.90, 128.54, 128.87, 133.36, 134.34, 135.18, 150.91, 151.94, 153.58 (14C_{ar}), 153.58 ($C_{oxadiazole}$), 166.34 (C_{ar}), 167.55 (C=O). Anal. calc. for $C_{20}H_{17}N_5O_4$ (391.38) (%): C 61.38; H 4.38; N 17.89. Found: C 59.15; H 4.39; N 17.80.

1-{2-[5-(3-chloro-4-methoxyphenyl)furan-3-yl]-5-(3-methyl-4-nitrophenyl)-1,3,4-oxadiazol-3(2*H*)-yl}ethanone (**26**)

Light brown powder, Yield: 31%, M.p.: 106°C (EtOH:acetone 3:1), IR: 3030 (CH, arom.), 2970 (CH, aliph.), 1691 (C=O), 1587 (C=N), 1256, 1042 (C-OC); ^{1}H NMR (600 MHz, DMSO-d_6): 2.09 (s, 3H, CH_3), 2.55 (s, 3H, CH_3), 3.94 (s, 3H, OCH_3), 7.26 (s, 1H, $CH_{oxadiazole}$), 7.28-7.29 (d, 2H, ArH, J = 6 Hz), 7.30-7.32 (d, 2H, ArH, J = 12 Hz), 7.65-7.66 (d, 1H, ArH, J = 6 Hz), 7.83-7.84 (d, 1H, ArH, J = 6 Hz), 7.84-7.85 (d, 1H, ArH, J = 6 Hz), 7.97-7.98 (d, 1H, ArH, J = 6 Hz); ^{13}C NMR (150 MHz, DMSO-d_6): 19.52 (CH_3), 21.04 (CH_3), 56.88 (OCH_3), 93.97 ($CH_{oxadiazole}$), 108.74, 113.89, 122.43, 122.67, 125.05, 125.71, 126.02, 126.75, 126.95, 128.54, 132.30, 133.31, 133.96, 134.99 (14C_{ar}), 151.98 ($C_{oxadiazole}$), 155.99, 157.47 (2C_{ar}), 166.30 (C=O). Anal. calc. for $C_{22}H_{18}ClN_3O_6$ (455.85) (%): C 57.97; H 3.98; N 9.22. Found: C 55.99; H 4.01; N 9.30.

1-[2-(1*H*-imidazol-4-yl)-5-(3-methyl-4-nitrophenyl)-1,3,4-oxadiazol-3(2*H*)-yl]ethanone (**27**)

Grey powder, Yield: 35%, M.p.: 166°C (EtOH:acetone 3:1), IR: 3016 (CH, arom.), 2970 (CH, aliph.), 1641 (C=O), 1587 (C=N), 1228, 1100 (C-OC); ^{1}H NMR (600 MHz, DMSO-d_6): 1.93 (s, 3H, CH_3), 2.56 (s, 3H, CH_3), 7.87-7.89 (m, 1H, ArH), 7.92-7.99 (m, 2H, ArH), 8.01-8.04 (m, 1H, ArH), 8.08-8.09 (d, 1H, ArH, J = 6 Hz), 10.01 (s, 1H, $CH_{oxadiazole}$), 10.55 (s, 1H, NH); ^{13}C NMR (150 MHz, DMSO-d_6): 19.77 (CH_3), 21.05 (CH_3), 90.67 ($CH_{oxadiazole}$), 125.05, 126.75, 128.39, 132.31, 133.32, 136.81, 138.72, 140.22, 150.50 (9C_{ar}), 151.15 ($C_{oxadiazole}$)), 168.87 (C=O). Anal. calc. for $C_{14}H_{13}N_5O_4$ (315.28) (%): C 53.33; H 4.16; N 22.21. Found: C 54.45; H 4.19; N 22.52.

1-{2-[4-(morpholin-4-yl)phenyl]-5-(3-methyl-4-nitrophenyl)-1,3,4-oxadiazol-3(2*H*)-yl]ethanone (**28**)

Light brown powder, Yield: 10%, M.p.: 140°C (EtOH:acetone 3:1), IR: 3034 (CH, arom.), 2836 (CH, aliph.), 1686 (C=O), 1515 (C=N), 1218, 1050 (C-OC); ^{1}H NMR (600 MHz, DMSO-d_6): 2.28 (s, 3H, CH_3), 2.57 (s, 3H, CH_3), 3.13-3.15 (m, 4H, 2x$CH_{2\text{-morpholine}}$), 3.72-3.74 (m, 4H, 2x$CH_{2\text{-morpholine}}$), 6,97-6,98 (d, 2H, ArH, J = 6 Hz), 7.15 (s, 1H, $CH_{oxadiazole}$), 7.31-7.32 (d, 2H, ArH, J = 6 Hz), 7.86-7.87 (m, 1H, ArH), 7.91-7.92 (m, 1H, ArH), 8.10-8.11 (d, 1H, ArH, J = 6 Hz); ^{13}C NMR (150 MHz, DMSO-d_6): 19.71 (CH_3), 21.76 (CH_3), 48.36 (2x$CH_{2\text{-morpholine}}$), 66.43 (2x$CH_{2\text{-morpholine}}$), 93.39 ($CH_{oxadiazole}$), 115.14, 125.76, 126.90, 128.06, 128.79, 128.93, 130.89, 134.30 (10C_{ar}), 150.83 ($C_{oxadiazole}$), 152.67, 153.48 (2C_{ar}), 167.19 (C=O). Anal. calc. for $C_{21}H_{22}N_4O_5$ (410.42) (%): C 61.45; H 5.40; N 13.65. Found: C 59.15; H 5.51; N 13.52.

1-{2-[4-(pyridin-2-yl)phenyl]-5-(3-methyl-4-nitrophenyl)-1,3,4-oxadiazol-3(2*H*)-yl]ethanone (**29**)

Yellow powder, Yield: 10%, M.p.: 160°C (EtOH:acetone 3:1), IR: 3016 (CH, arom.), 2970 (CH, aliph.), 1738 (C=O), 1519 (C=N), 1228, 1075 (C-OC); ^{1}H NMR (600 MHz, DMSO-d_6): 2.32 (s, 3H, CH_3), 2.66 (s, 3H, CH_3), 7.31 (s, 1H, $CH_{oxadiazole}$), 7.38-7.47 (m, 1H, ArH), 7.95-7.99 (m, 1H, ArH), 8.05-8.07 (m, 1H, ArH), 8.13-8.16 (m, 1H, ArH), 8.22-8.23 (m, 1H, ArH), 8.30-8.32 (d, 2H, ArH, J = 12 Hz), 8.33-8.34 (m, 1H, ArH), 8.38-8.39 (d, 2H, ArH, J = 6 Hz), 8.68-8.76 (m, 1H, ArH); ^{13}C NMR (150 MHz, DMSO-d_6): 19.76 (CH_3), 21.74 (CH_3), 92.91 ($CH_{oxadiazole}$), 121.36, 123.81, 123.98, 126.06, 127.04, 127.46, 127.85, 128.47, 130.23, 131.26, 134.59, 137.99, 142.47, 150.28 (16C_{ar}), 151.08 ($C_{oxadiazole}$), 154.98 (C_{ar}), 165.03 (C=O). Anal. calc. for $C_{22}H_{18}N_4O_4$ (402.40) (%): C 65.66; H 4.51; N 13.92. Found: C 65.99; H 4.51; N 13.58.

1-[5-(3-methyl-4-nitrophenyl)-2-(1*H*-pyrrol-2-yl)-1,3,4-oxadiazol-3(2*H*)-yl]ethanone (**30**)

Dark brown powder, Yield: 10%, M.p.: 200°C (EtOH:acetone 3:1), IR: 3040 (CH, arom.), 2850 (CH, aliph.), 1686 (C=O), 1586 (C=N), 1269, 1034 (C-OC); ^{1}H NMR (600 MHz, DMSO-d_6): 2.39 (s, 3H, CH_3), 2.62 (s, 3H, CH_3), 7.22 (s, 1H, $CH_{oxadiazole}$), 7.94-7.96 (m, 2H, ArH), 8.04-8.05 (m, 2H, ArH), 8.06-8.07 (m, 1H, ArH), 8.07-8.08 (m, 1H, ArH), 13.55 (s, 1H, NH); ^{13}C NMR (150 MHz, DMSO-d_6): 19.53 (CH_3), 21.01 (CH_3), 93.48 ($CH_{oxadiazole}$), 125.02, 126.75, 128.51, 132.30, 133.23, 133.93, 135.27, 136.81, 151.14 (9C_{ar}), 151.88

($C_{oxadiazole}$), 164.37 (C_{ar}), 166.36 (C=O). Anal. calc. for $C_{15}H_{14}N_4O_4$ (314.30) (%): C 57.32; H 4.49; N 17.83. Found: C 57.15; H 4.51; N 17.52.

1-{5-(3-methyl-4-nitrophenyl)-2-[4-(pyrrolidin-1-yl)phenyl]-1,3,4-oxadiazol-3(2*H*)-yl}ethanone (**31**)

Dark brown powder, Yield: 10%, M.p.: 102°C (EtOH:acetone 3:1), IR: 3021 (CH, arom.), 2969 (CH, aliph.), 1738 (C=O), 1518 (C=N), 1228, 1158 (C-OC); ^{1}H NMR (600 MHz, DMSO-d_6): 1.97-1.99 (m, 4H, 2x$CH_{2\text{-}pyrrolidine}$), 2.30 (s, 3H, CH_3), 2.39-2.40 (m, 2H, $CH_{2\text{-}pyrrolidine}$), 2.55 (s, 3H, CH_3), 2.62-2.63 (m, 2H, $CH_{2\text{-}pyrrolidine}$), 6.63-6.65 (d, 2H, ArH, J = 12 Hz), 7.67-7.69 (d, 2H, ArH, J = 12 Hz), 7.95-7.96 (m, 1H, ArH), 8.04-8.06 (m, 2H, ArH), 9.65 (s, 1H, $CH_{oxadiazole}$); ^{13}C NMR (150 MHz, DMSO-d_6): 19.77 (CH_3), 21.04 (CH_3), 25.38 (2x$CH_{2\text{-}pyrrolidine}$), 47.83 (2x$CH_{2\text{-}pyrrolidine}$), 111.74 ($CH_{oxadiazole}$), 124.71, 125.05, 126.75, 128.50, 132.31, 133.31, 133.92, 151.15 (10C_{ar}), 152.14 ($C_{oxadiazole}$), 164.38, 166.39 (2C_{ar}), 168.88 (C=O). Anal. calc. for $C_{21}H_{22}N_4O_4$ (394.42) (%): C 63.95; H 5.62; N 14.20. Found: C 59.15; H 5.51; N 14.28.

1-{5-(3-methyl-4-nitrophenyl)-2-[4-(piperidin-1-yl)phenyl]-1,3,4-oxadiazol-3(2*H*)-yl}ethanone (**32**)

Violet powder, Yield: 10%, M.p.: 190°C (EtOH:acetone 3:1), IR: 3021 (CH, arom.), 2948 (CH, aliph.), 1682 (C=O), 1515 (C=N), 1257, 1017 (C-OC); ^{1}H NMR (600 MHz, DMSO-d_6): 1.56-1.62 (m, 4H, 2x$CH_{2\text{-}piperidine}$), 1.93 (s, 3H, CH_3), 2.39-2.40 (m, 2H, $CH_{2\text{-}piperidine}$), 2.56 (s, 3H, CH_3), 2.61-2.62 (m, 2H, $CH_{2\text{-}piperidine}$), 3.42-3.44 (m, 2H, $CH_{2\text{-}piperidine}$), 7.01-7.03 (d, 2H, ArH, J = 12 Hz), 7.67-7.69 (d, 2H, ArH, J = 12 Hz), 7.84-7.89 (m, 2H, ArH), 7.95-7.97 (m, 1H, ArH), 9.68 (s, 1H, $CH_{oxadiazole}$); ^{13}C NMR (75 MHz, DMSO-d_6): 19.78 (CH_3), 21.04 (CH_3), 24.43 ($CH_{2\text{-}piperidine}$), 25.31 (2x$CH_{2\text{-}piperidine}$), 48.07 (2x$CH_{2\text{-}piperidine}$), 113.49 ($CH_{oxadiazole}$), 125.07, 125.88, 126.75, 128.54, 132.10, 132.32, 133.34, 136.80, 151.14 (11C_{ar}), 155.08 ($C_{oxadiazole}$), 164.38 (C_{ar}), 168.90 (C=O). Anal. calc. for $C_{22}H_{24}N_4O_4$ (408.45) (%): C 64.69; H 5.92; N 13.72. Found: C 59.95; H 5.61; N 13.62.

1-[5-(3-methyl-4-nitrophenyl)-2-(pyridin-2-yl)-1,3,4-oxadiazol-3(2*H*)-yl]ethanone (**33**)

Yellow powder, Yield: 11%, M.p.: 86°C (EtOH:acetone 3:1), IR: 3015 (CH, arom.), 2970 (CH, aliph.), 1654 (C=O), 1580 (C=N), 1288, 1074 (C-OC); ^{1}H NMR (600 MHz, DMSO-d_6): 2.28 (s, 3H, CH_3), 2.57 (s, 3H, CH_3), 7.23 (s, 1H, $CH_{oxadiazole}$), 7.47-7.49 (m, 1H, ArH), 7.66-7.67 (d, 1H, ArH, J = 6 Hz), 7.87-7.88 (m, 1H, ArH), 7.91-7.94 (m, 2H, ArH), 8.10-8.11 (d,

1H, ArH, *J* = 6 Hz), 8.62-8.63 (m, 1H, ArH); ^{13}C NMR (75 MHz, DMSO-*d₆*): 19.72 (CH_3), 21.53 (CH_3), 92.85 ($CH_{oxadiazole}$), 123.14, 125.61, 125.76, 125.79, 128.59, 130.90, 134.38, 137.84, 150.53, 150.81, 153.71 ($11C_{ar}$), 154.30 ($C_{oxadiazole}$), 166.95 (C=O). Anal. calc. for $C_{16}H_{14}N_4O_4$ (326.31) (%): C 58.89; H 4.32; N 17.17. Found: C 61.15; H 4.35; N 17.52.

1-[5-(3-methyl-4-nitrophenyl)-2-(pyridin-3-yl)-1,3,4-oxadiazol-3(2*H*)-yl]ethanone (**34**)

Dark brown powder, Yield: 22%, M.p.: 110°C (EtOH:acetone 3:1), IR: 3016 (CH, arom.), 2970 (CH, alif.), 1738 (C=O), 1513 (C=N), 1229, 1063 (C-OC); ^{1}H NMR (600 MHz, DMSO-*d*$_6$): 2.30 (s, 3H, CH_3), 2.57 (s, 3H, CH_3), 7.34 (s, 1H, $CH_{oxadiazole}$), 7.49-7.51 (m, 1H, ArH), 7.90-7.92 (m, 3H, ArH), 7.94-7.95 (m, 1H, ArH), 8.11-8.13 (d, 1H, ArH, *J* = 12 Hz), 8.66-8.68 (m, 1H, ArH); ^{13}C NMR (150 MHz, DMSO-*d*$_6$): 19.66 (CH_3), 21.68 (CH_3), 91.43 ($CH_{oxadiazole}$), 124.58, 125.73, 125.92, 128.25, 131.06, 133.73, 134.27, 134.98, 148.70, 150.96, 151.57 ($11C_{ar}$), 153.62 ($C_{oxadiazole}$), 167.70 (C=O). Anal. calc. for $C_{16}H_{14}N_4O_4$ (326.31) (%): C 58.89; H 4.32; N 17.17. Found: C 61.15; H 4.35; N 17.52.

1-[5-(3-methyl-4-nitrophenyl)-2-(thiophen-2-yl)-1,3,4-oxadiazol-3(2*H*)-yl]ethanone (**35**)

Yellowish powder, Yield: 12%, M.p.: 196°C (EtOH:acetone 3:1), IR: 3030 (CH, arom.), 2977 (CH, aliph.), 1640 (C=O), 1518 (C=N), 1282, 1099 (C-OC); ^{1}H NMR (600 MHz, DMSO-*d*$_6$): 2.39 (s, 3H, CH_3), 2.55 (s, 3H, CH_3), 7.95-7.96 (m, 1H, ArH), 7.96-7.97 (m, 1H, ArH), 8.03-8.04 (m, 3H, ArH), 8.05-8.06 (m, 1H, ArH), 8.06 (s, 1H, $CH_{oxadiazole}$); ^{13}C NMR (150 MHz, DMSO-*d*$_6$): 19.52 (CH_3), 21.04 (CH_3), 125.05 ($CH_{oxadiazole}$), 126.76, 128.54, 132.32, 133.27, 133.32, 133.96, 136.83, 151.16 ($8C_{ar}$), 151.95 ($C_{oxadiazole}$), 164.39, 166.34 ($2C_{ar}$) 168.89 (C=O). Anal. calc. for $C_{15}H_{13}N_3O_4S$ (331.35) (%): C 54.37; H 3.95; N 12.68. Found: C 51.15; H 4.15; N 12.92.

1-[2-(5-chlorofuran-2-yl)-5-(3-methyl-4-nitrophenyl)-1,3,4-oxadiazol-3(2*H*)-yl]ethanone (**36**)

Brown powder, Yield: 21%, M.p.: 106°C (EtOH:acetone 3:1), IR: 3037 (CH, arom.), 2978 (CH, aliph.), 1643 (C=O), 1518 (C=N), 1281, 1071 (C-OC); ^{1}H NMR (600 MHz, DMSO-*d*$_6$): 2.17 (s, 3H, CH_3), 2.59 (s, 3H, CH_3), 6.20-6.31 (d, 1H, ArH, *J* = 6 Hz), 7.39 (s, 1H, $CH_{oxadiazole}$), 7.94-7.97 (m, 1H, ArH), 8.03-8.07 (m, 2H, ArH), 8.17-8.18 (m, 1H, ArH); ^{13}C NMR (150 MHz, DMSO-*d*$_6$): 19.79 (CH_3), 21.04 (CH_3), 125.08 ($CH_{oxadiazole}$), 126.75, 128.55, 132.31, 133.30, 133.97, 134.98, 136.80, 151.14 ($8C_{ar}$), 151,97 ($C_{oxadiazole}$), 164.38, 166.32

(2C_{ar}), 168.89 (C=O). Anal. calc. for $C_{15}H_{12}ClN_3O_5$ (349.73) (%): C 51.51; H 3.46; N 12.02. Found: C 53.22; H 3.35; N 12.52.

1-[5-(3-methyl-4-nitrophenyl)-2-(quinolin-4-yl)-1,3,4-oxadiazol-3(2*H*)-yl]ethanone (**37**)
Grey powder, Yield: 23%, M.p.: 134°C (EtOH:acetone 3:1), IR: 3024 (CH, arom.), 2359 (CH, aliph.), 1664 (C=O), 1516 (C=N), 1240, 1070 (C-OC); ^{1}H NMR (600 MHz, DMSO-d_6): 2.41 (s, 3H, CH_3), 2.54 (s, 3H, CH_3), 7.57-7.58 (d, 1H, ArH, J = 6 Hz), 7.78-7.80 (m, 1H, ArH), 7.86-7.91 (m, 2H, ArH), 8.03 (s, 1H, $CH_{oxadiazole}$), 8.08-8.09 (d, 1H, ArH, J = 6 Hz), 8.15-8.17 (d, 1H, ArH, J = 12 Hz), 8.34-8.36 (d, 1H, ArH, J = 12 Hz), 8.39-8.40 (d, 1H, ArH, J = 6 Hz), 8.99-9.00 (d, 1H, ArH, J = 6 Hz); ^{13}C NMR (150 MHz, DMSO-d_6): 19.61 (CH_3), 21.70 (CH_3), 90.23 ($CH_{oxadiazole}$), 119.51, 121.98, 123.81, 125.13, 125.77, 125.93, 128.23, 130.34, 130.39, 130.45, 130.99, 134.32, 139.61, 148.63, 151.13 (15C_{ar}), 153.81 ($C_{oxadiazole}$), 168.12 (C=O). Anal. calc. for $C_{20}H_{16}N_4O_4$ (376.37) (%): C 63.82; H 4.28; N 14.89. Found: C 61.45; H 4.31; N 14.72.

Table S1. The activity data of compounds **2-19** expressed as MIC (MBC or MFC) [µg/mL] and {MBC/MIC or MFC/MIC} values against the reference strains of microorganisms.

	Species / Compound No	MIC (MBC or MFC) [µg/ml] and {MBC/MIC or MFC/MIC} compounds and reference substances																			
		2	3	4	6	8	9	10	11	12	13	14	15	16	17	18	19	CIP/ NY*	NIT	CFX	APC
Gram-positive bacteria	*Staphylococcus aureus* ATCC 25923	31.25<br>(62.5)<br>{2}	500<br>(>1000)<br>{>1}	250<br>(>1000)<br>{>4}	1000<br>(>1000)<br>{>1}	250<br>(>1000)<br>{>4}	1000<br>(>1000)<br>{>1}	250<br>(>1000)<br>{>4}	500<br>(>1000)<br>{>2}	1000<br>(>1000)<br>{>1}	125<br>(>1000)<br>{>8}	250<br>(>1000)<br>{>4}	125<br>(>1000)<br>{>8}	500<br>(>1000)<br>{>2}	-	500<br>(1000)<br>{2}	500<br>(1000)<br>{2}	0.48<br>(0.48)<br>{1}	15.62<br>(15.62)	0.49	nd
	Staphylococcus aureus ATCC 6538	62.5<br>(125)<br>{2}	-	500<br>(>1000)<br>{>2}	-	500<br>(>1000)<br>{>2}	1000<br>(>1000)<br>{>1}	1000<br>(>1000)<br>{>1}	500<br>(>1000)<br>{>2}	-	250<br>(>1000)<br>{>4}	250<br>(>1000)<br>{>4}	1000<br>(>1000)<br>{>1}	500<br>(>1000)<br>{>2}	-	500<br>(1000)<br>{2}	500<br>(1000)<br>{2}	0.24<br>(0.24)<br>{1}	15.62<br>(15.62)	0.98	nd
	Staphylococcus aureus ATCC 43300	125<br>(500)<br>{4}	-	500<br>(>1000)<br>{>2}	500<br>(>1000)<br>{>2}	500<br>(>1000)<br>{>2}	-	-	500<br>(>1000)<br>{>2}	-	1000<br>(>1000)<br>{>1}	1000<br>(>1000)<br>{>1}	1000<br>(>1000)<br>{>1}	500<br>(>1000)<br>{>2}	-	500<br>(1000)<br>{2}	500<br>(1000)<br>{2}	0.24<br>(0.24)<br>{1}	7.81<br>(15.62)	nd	nd
	Staphylococcus aureus ATCC 29213	125<br>(500)<br>{4}	1000<br>(>1000)<br>{>1}	500<br>(>1000)<br>{>2}	1000<br>(>1000)<br>{>1}	500<br>(>1000)<br>{>2}	-	500<br>(>1000)<br>{>2}	500<br>(>1000)<br>{>2}	-	500<br>(>1000)<br>{>2}	500<br>(>1000)<br>{>2}	1000<br>(>1000)<br>{>1}	1000<br>(>1000)<br>{>1}	-	500<br>(1000)<br>{2}	500<br>(1000)<br>{2}	0.48<br>(0.48)<br>{1}	nd	nd	nd
	Staphylococcus epidermidis ATCC 12228	15.62<br>(15.62)<br>{1}	500<br>(>1000)<br>{>1}	250<br>(>1000)<br>{>4}	1000<br>(>1000)<br>{>1}	500<br>(>1000)<br>{>2}	250<br>(>1000)<br>{>4}	250<br>(>1000)<br>{>4}	250<br>(>1000)<br>{>4}	-	500<br>(>1000)<br>{>2}	250<br>(>1000)<br>{>4}	500<br>(>1000)<br>{>2}	500<br>(>1000)<br>{>2}	1000<br>(>1000)<br>{>1}	500<br>(1000)<br>{2}	500<br>(1000)<br>{2}	0.12<br>(0.12)<br>{1}	3.91<br>(7.81)	0.24	nd
	Enterococcus faecalis ATCC 29212	1000<br>(>1000)<br>{>1}	-	1000<br>(>1000)<br>{>1}	1000<br>(>1000)<br>{>1}	500<br>(>1000)<br>{>2}	-	1000<br>(>1000)<br>{>1}	500<br>(>1000)<br>{>1}	-	500<br>(>1000)<br>{>2}	500<br>(>1000)<br>{>2}	1000<br>(>1000)<br>{>1}	1000<br>(>1000)<br>{>1}	-	500<br>(1000)<br>{2}	500<br>(1000)<br>{2}	0.98<br>(1.95)<br>{2}	nd	nd	nd
	Micrococcus luteus ATCC 10240	1000<br>(>1000)<br>{>1}	250<br>(>1000)<br>{>1}	125<br>(>1000)<br>{>8}	500<br>(>1000)<br>{>2}	500<br>(>1000)<br>{>2}	1000<br>(>1000)<br>{>1}	250<br>(>1000)<br>{>4}	250<br>(>1000)<br>{>4}	500<br>(>1000)<br>{>2}	250<br>(>1000)<br>{>4}	31.25<br>(>1000)<br>{>32}	500<br>(>1000)<br>{>2}	250<br>(>1000)<br>{>2}	250<br>(>1000)<br>{>4}	500<br>(1000)<br>{2}	500<br>(1000)<br>{2}	0.98<br>(1.95)<br>{2}	62.5<br>(62.5)	0.98	nd
	Bacillus subtilis ATCC 6633	62.5<br>(125)<br>{2}	-	500<br>(>1000)<br>{>2}	1000<br>(>1000)<br>{>1}	500<br>(>1000)<br>{>2}	-	1000<br>(>1000)<br>{>1}	125<br>(>1000)<br>{>8}	1000<br>(>1000)<br>{>1}	250<br>(>1000)<br>{>4}	31.25<br>(>1000)<br>{>32}	125<br>(>1000)<br>{>8}	-	-	500<br>(500)<br>{1}	500<br>(500)<br>{1}	0.03<br>(0.03)<br>{1}	3.91<br>(3.91)	15.62	62.5
	Bacillus cereus ATCC 10876	1000<br>(>1000)<br>{>1}	-	1000<br>(>1000)<br>{>1}	1000<br>(>1000)<br>{>1}	500<br>(>1000)<br>{>2}	-	-	500<br>(>1000)<br>{>2}	1000<br>(>1000)<br>{>1}	500<br>(>1000)<br>{>2}	1000<br>(>1000)<br>{>1}	1000<br>(>1000)<br>{>1}	-	-	500<br>(1000)<br>{2}	500<br>(1000)<br>{2}	0.06<br>(0.12)<br>{2}	7.81<br>(15.62)	31.25	nd

**Gram-negative bacteria**	*Bordetella bronchiseptica* ATCC 4617	-	-	1000<br>(>1000)<br>**{>1}**	-	-	-	-	500<br>(>1000)<br>**{>2}**	1000<br>(>1000)<br>**{>1}**	-	-	-	-	-	500<br>(1000)<br>**{2}**	500<br>(1000)<br>**{2}**	0.98<br>(0.98)<br>{1}	125<br>(>1000)	nd	nd
	Klebsiella pneumoniae ATCC 13883	-	-	-	-	-	-	-	1000<br>(>1000)<br>**{>1}**	-	-	-	-	-	-	1000<br>(>1000)<br>**{>1}**	1000<br>(>1000)<br>**{>1}**	0.12<br>(0.24)<br>{2}	15.62<br>(31.25)	nd	nd
	Proteus mirabilis ATCC 12453	-	-	-	-	-	-	-	1000<br>(>1000)<br>**{>1}**	-	-	-	-	-	-	-	-	0.03<br>(0.03)<br>{1}	62.5<br>(125)	nd	nd
	Salmonella typhimurium ATCC 14028	-	-	-	-	-	-	-	500<br>(>1000)<br>**{>2}**	-	-	-	1000<br>(>1000)<br>**{>1}**	-	-	1000<br>(>1000)<br>**{>1}**	1000<br>(>1000)<br>**{>1}**	0.06<br>(0.06)<br>{1}	31.25<br>(62.5)	nd	nd
	Escherichia coli ATCC 25922	-	-	-	-	-	-	-	500<br>(>1000)<br>**{>2}**	-	-	-	1000<br>(>1000)<br>**{>1}**	-	-	1000<br>(1000)<br>**{1}**	1000<br>(1000)<br>**{1}**	0.004<br>(0.008)<br>{2}	7.81<br>(15.62)	nd	nd
	Pseudomonas aeruginosa ATCC 9027	-	-	-	-	-	-	-	-	-	-	-	1000<br>(>1000)<br>**{>1}**	-	-	-	-	0.48<br>(0.98)<br>{2}	-	nd	nd
**Fungi**	*Candida albicans* ATCC 2091	1000<br>(>1000)<br>**{>1}**	-	-	-	-	-	-	1000<br>(>1000)<br>**{>1}**	500<br>(1000)<br>**{2}**	-	-	-	-	-	125<br>(500)<br>**{4}**	125<br>(500)<br>**{4}**	0.24*<br>(0.24)<br>**{1}**	na	na	na
	Candida albicans ATCC 10231	1000<br>(>1000)<br>**{>1}**	-	500<br>(>1000)<br>**{>2}**	-	-	-	-	500<br>(>1000)<br>**{>2}**	500<br>(1000)<br>**{2}**	-	-	-	-	-	62.5<br>(250)<br>**{4}**	62,5<br>(250)<br>**{4}**	0.48*<br>(0.48)<br>**{1}**	na	na	na
	Candida parapsilosis ATCC 22019	1000<br>(>1000)<br>**{>1}**	-	1000<br>(>1000)<br>**{>1}**	-	-	-	-	500<br>(>1000)<br>**{>2}**	250<br>(500)<br>**{2}**	1000<br>(>1000)<br>**{>1}**	-	-	-	-	125<br>(250)<br>**{2}**	125<br>(250)<br>**{2}**	0.24*<br>(0.48)<br>{2}	na	na	na
	Candida glabrata ATCC 90030	-	-	-	-	-	-	-	1000<br>(>1000)<br>**{>1}**	500<br>(500)<br>**{1}**	-	-	-	-	-	250<br>(250)<br>**{1}**	250<br>(250)<br>**{1}**	0.24*<br>(0.48)<br>{2}	na	na	na
	Candida krusei ATCC 14243	1000<br>(>1000)<br>**{>1}**	-	-	-	-	-	-	-	250<br>(1000)<br>**{4}**	-	-	-	-	-	125<br>(250)<br>**{2}**	125<br>(250)<br>**{2}**	0.24*<br>(0.24)<br>{1}	na	na	na

"–" no activity; nd, not determined; na, not applicable; the standard chemotherapeutics used as positive controls: ciprofloxacin (CIP), nitrofurantoin (NIT), cefuroxime (CFX) and ampicillin (APC) for bacteria and nystatin (NY*), Compounds with bactericidal effect (MBC/MIC ≤4) or fungicidal effect (MFC/MIC ≤4), MBC/MIC or MFC/MIC in these

cases are bolded. no bioactivity – MIC > 1000 µg/ml ; mild bioactivity – MIC = 501 – 1000 µg/ml ; moderate bioactivity – MIC = 126 – 500 µg/ml ; good bioactivity – MIC = 26 – 125 µg/ml; strong bioactivity – MIC = 10 – 25 µg/ml ; very strong bioactivity – MIC < 10 µg/ml.

Table S2. The activity data of compounds **20**-**37** expressed as MIC (MBC or MFC) [µg/mL] and {MBC/MIC or MFC/MIC} values against the reference strains of microorganisms.

	Species / Compound No	MIC (MBC or MFC) [µg/ml] and {MBC/MIC or MFC/MIC} compounds and reference substances																					
		20	21	22	23	24	25	26	27	28	29	30	31	32	33	34	35	36	37	CIP/ VA*/ NY**	NIT	CFX	APC
Gram-positive bacteria	*Staphylococcus aureus* ATCC 25923	15.62<br>(15.62)<br>**{1}**	-	-	-	1000<br>(>2000)<br>**{>2}**	-	-	-	-	-	1000<br>(>2000/<br>**{>2}**	1000<br>(2000)<br>**{2}**	500<br>(1000)<br>**{2}**	-	-	-	-	7.81<br>(7.81)<br>**{1}**	0.48<br>(0.48)<br>{1}	15.62<br>(15.62)	0.49	nd
	Staphylococcus aureus ATCC 6538	15.62<br>(15.62)<br>**{1}**	1000<br>(2000)<br>**{2}**	1000<br>(2000)<br>**{2}**	-	1000<br>(>2000)<br>**{>2}**	125<br>(>2000)<br>{>16}	-	1000<br>(>2000)<br>**{>2}**	-	-	-	1000<br>(2000)<br>**{2}**	500<br>(2000)<br>**{4}**	500<br>(>2000)<br>{>4}	250<br>(>2000)<br>{>8}	1000<br>(>2000)<br>**{>2}**	1000<br>(2000)<br>**{2}**	7.81<br>(7.81)<br>**{1}**	0.24<br>(0.24)<br>{1}	15.62<br>(15.62)	0.98	nd
	Staphylococcus aureus ATCC 43300	15.62<br>(15.62)<br>**{1}**	-	-	-	1000<br>(>2000)<br>**{>2}**	-	-	-	-	-	-	-	1000<br>(2000)<br>**{2}**	-	-	-	-	15.62<br>(15.62)<br>**{1}**	0.24<br>(0.24)<br>{1}	7.81<br>(15.62)	nd	nd
	Staphylococcus aureus ATCC 29213	15.62<br>(15.62)<br>**{1}**	-	-	500<br>(>2000)<br>{>4}	500<br>(>2000)<br>{>4}	-	-	-	-	-	1000<br>(>2000)<br>**{>2}**	-	500<br>(500)<br>**{1}**	-	1000<br>(>2000)<br>**{>2}**	-	-	15.62<br>(15.62)<br>**{1}**	0.48<br>(0.48)<br>{1}	nd	nd	nd
	Staphylococcus epidermidis ATCC 12228	1.95<br>(3.91)<br>**{2}**	7.81<br>(15.62)<br>**{2}**	15.62<br>(62.5)<br>**{4}**	62.5<br>(500)<br>{8}	250<br>(250)<br>**{1}**	62.5<br>(125)<br>**{2}**	31.25<br>(31.25)<br>**{1}**	62.5<br>(250)<br>**{2}**	31.25<br>(125)<br>**{4}**	1000<br>(>2000)<br>**{>2}**	7.81<br>(15.62)<br>**{2}**	31.25<br>(125)<br>**{4}**	62.5<br>(125)<br>**{2}**	1000<br>(2000)<br>**{2}**	62.5<br>(1000)<br>{16}	15.62<br>(15.62)<br>**{1}**	31.25<br>(62.5)<br>**{2}**	0.48<br>(0.48)<br>**{1}**	0.12<br>(0.12)<br>{1}	3.91<br>(7.81)	0.24	nd
	Enterococcus faecalis ATCC 29212	125<br>(500)<br>**{4}**	-	1000<br>(>2000)<br>**{>2}**	250<br>(>2000)<br>{>8}	500<br>(>2000)<br>{>4}	-	-	-	-	-	1000<br>(>2000)<br>**{>2}**	-	500<br>(>2000)<br>{>4}	-	1000<br>(>2000)<br>**{>2}**	-	-	500<br>(1000)<br>**{2}**	0.98*<br>(1.95)<br>{2}	nd	nd	nd
	Micrococcus luteus ATCC 10240	125<br>(250)<br>**{2}**	1000<br>(2000)<br>**{2}**	1000<br>(2000)<br>**{2}**	-	1000<br>(2000)<br>**{2}**	250<br>(2000)<br>{8}	-	1000<br>(2000)<br>**{2}**	-	-	1000<br>(>2000)<br>**{>2}**	500<br>(1000)<br>**{2}**	250<br>(500)<br>**{2}**	500<br>(2000)<br>{4}	500<br>(2000)<br>**{4}**	-	1000<br>(1000)<br>{1}	62.5<br>(62.5)<br>**{1}**	0.98<br>(1.95)<br>{2}	62.5<br>(62.5)	0.98	nd
	Bacillus subtilis ATCC 6633	31.25<br>(62.5)<br>**{2}**	1000<br>(2000)<br>**{1}**	1000<br>(2000)<br>**{2}**	250<br>(>2000)<br>{>8}	125<br>(2000)<br>{16}	-	500<br>(2000)<br>**{4}**	-	-	-	1000<br>(2000)<br>**{2}**	-	1000<br>(2000)<br>**{2}**	-	-	-	1000<br>(2000)<br>**{2}**	31.25<br>(31.25)<br>**{1}**	0.03<br>(0.03)<br>{1}	3.91<br>(3.91)	15.62	62.5
	Bacillus cereus ATCC 10876	31.25<br>(31.25)<br>**{1}**	1000<br>(>2000)<br>**{>2}**	1000<br>(>2000)<br>**{>2}**	250<br>(>2000)<br>{>8}	125<br>(>2000)<br>{>16}	-	500<br>(>2000)<br>{>4}	-	-	-	1000<br>(>2000)<br>**{>2}**	1000<br>(>2000)<br>**{>2}**	500<br>(>2000)<br>{>4}	-	1000<br>(>2000)<br>**{>2}**	-	-	31.25<br>(62.5)<br>**{2}**	0.06<br>(0.12)<br>{2}	7.81<br>(15.62)	31.25	nd

Gram-negative bacteria	*Bordetella bronchiseptica* ATCC 4617	125<br>(500)<br>**{4}**	-	1000<br>(2000)<br>**{2}**	-	1000<br>(>2000)<br>**{>2}**	-	-	1000<br>(2000)<br>**{2}**	-	-	1000<br>(2000)<br>**{2}**	1000<br>(1000)<br>**{1}**	1000<br>(2000)<br>**{2}**	-	-	-	-	1000<br>(2000)<br>**{2}**	0.98<br>(0.98)<br>{1}	125<br>(>1000)	nd	nd
	Klebsiella pneumoniae ATCC 13883	125<br>(500)<br>**{4}**	-	-	-	500<br>(>2000)<br>{>4}	-	-	-	-	-	-	-	-	-	-	-	-	-	0.12<br>(0.24)<br>{2}	15.62<br>(31.25)	nd	nd
	Proteus mirabilis ATCC 12453	125<br>(500)<br>**{4}**	-	-	-	500<br>(>2000)<br>{>4}	-	-	-	-	-	-	-	-	-	-	-	-	-	0.03<br>(0.03)<br>{1}	62.5<br>(125)	nd	nd
	Salmonella typhimurium ATCC 14028	125<br>(500)<br>**{4}**	-	-	-	500<br>(>2000)<br>{>4}	-	-	-	-	-	-	-	-	-	-	-	-	1000<br>(>2000)<br>**{>2}**	0.06<br>(0.06)<br>{1}	31.25<br>(62.5)	nd	nd
	Escherichia coli ATCC 25922	62.5<br>(125)<br>**{2}**	-	-	-	250<br>(>2000)<br>{>8}	-	-	-	-	-	-	-	-	-	-	-	-	1000<br>(1000)<br>**{1}**	0.004<br>(0.008)<br>{2}	7.81<br>(15.62)	nd	nd
	Pseudomonas aeruginosa ATCC 9027	1000<br>(2000)<br>**{2}**	-	-	-	-	-	-	-	-	-	-	-	-	-	-	-	-	-	0.48<br>(0.98)<br>{2}	-	nd	nd
Fungi	*Candida albicans* ATCC 2091	31.25<br>(62.5)<br>**{2}**	-	1000<br>(2000)<br>**{2}**	-	250<br>(2000)<br>{8}	1000<br>(2000)<br>**{2}**	-	-	1000<br>(2000)<br>**{2}**	1000<br>(2000)<br>**{2}**	1000<br>(2000)<br>**{2}**	250<br>(500)<br>**{2}**	500<br>(500)<br>**{1}**	-	1000<br>(2000)<br>**{2}**	-	-	15.62<br>(15.62)<br>**{1}**	0.24**<br>(0.24)<br>{1}	na	na	na
	Candida albicans ATCC 10231	125<br>(250)<br>**{2}**	-	1000<br>(2000)<br>**{2}**	-	125<br>(2000)<br>{16}	-	-	-	-	1000<br>(>2000)<br>**{>2}**	-	500<br>(1000)<br>**{2}**	500<br>(500)<br>**{1}**	1000<br>(2000)<br>**{2}**	500<br>(2000)<br>**{4}**	-	-	62.5<br>(500)<br>{8}	0.48**<br>(0.48)<br>{1}	na	na	na
	Candida parapsilosis ATCC 22019	125<br>(500)<br>**{4}**	-	-	-	62.5<br>(1000)<br>{16}	1000<br>(2000)<br>**{2}**	500<br>(2000)<br>**{4}**	-	1000<br>(2000)<br>**{2}**	-	1000<br>(2000)<br>**{2}**	500<br>(500)<br>**{1}**	500<br>(500)<br>**{1}**	1000<br>(2000)<br>**{2}**	500<br>(2000)<br>**{4}**	-	-	250<br>(2000)<br>{8}	0.24**<br>(0.48)<br>{2}	na	na	na
	Candida glabrata ATCC 90030	62.5<br>(62,5)<br>**{1}**	-	-	-	125<br>(250)<br>**{2}**	-	500<br>(2000)<br>**{4}**	-	-	-	-	500<br>(1000)<br>**{2}**	500<br>(1000)<br>**{2}**	-	1000<br>(2000)<br>**{2}**	-	-	500<br>(2000)<br>**{4}**	0.24**<br>(0.48)<br>{2}	na	na	na
	Candida krusei ATCC 14243	62.5<br>(250)<br>**{4}**	-	-	-	250<br>(2000)<br>{8}	-	-	-	-	-	-	500<br>(500)<br>**{1}**	500<br>(500)<br>**{1}**	-	1000<br>(1000)<br>**{1}**	-	-	500<br>(2000)<br>**{4}**	0.24**<br>(0.24)<br>{1}	na	na	na

"–" no activity; nd, not determined; na, not applicable; the standard chemotherapeutics used as positive controls: ciprofloxacin (CIP), nitrofurantoin (NIT), cefuroxime (CFX), vancomycin (VA*) and ampicillin (APC) against bacteria and nystatin (NY**) for fungi. Compounds with bactericidal effect (MBC/MIC ≤4) or fungicidal effect (MFC/MIC ≤4), MBC/MIC or MFC/MIC in these cases are bolded. no bioactivity – MIC > 1000 μg/ml ; mild bioactivity – MIC = 501 – 1000 μg/ml ; moderate bioactivity – MIC = 126 – 500 μg/ml ; good bioactivity – MIC = 26 – 125 μg/ml; strong bioactivity – MIC = 10 – 25 μg/ml ; very strong bioactivity – MIC < 10 μg/ml.

FIGURES

Examples of ^{1}H NMR and ^{13}C NMR spectra of synthesized hydrazide-hydrazones and 3-acetyl-2,5-disubstituted-1,3,4-oxadiazolines:

Figure S1. ^{1}H NMR spectrum of compound **2**

Figure S2. ^{13}C NMR spectrum of compound **2**

Figure S3. ^{1}H NMR spectrum of compound **18**

Figure S4. ^{13}C NMR spectrum of compound **18**

Figure S5. ^{1}H NMR spectrum of compound **23**

Figure S6. ^{13}C NMR spectrum of compound **23**

Figure S7. ^{1}H NMR spectrum of compound **33**

Figure S8. ^{13}C NMR spectrum of compound **33**

Compound **2**: *N*-[(5-iodofuran-2-yl)methylidene]-3-methyl-4-nitrobenzohydrazide

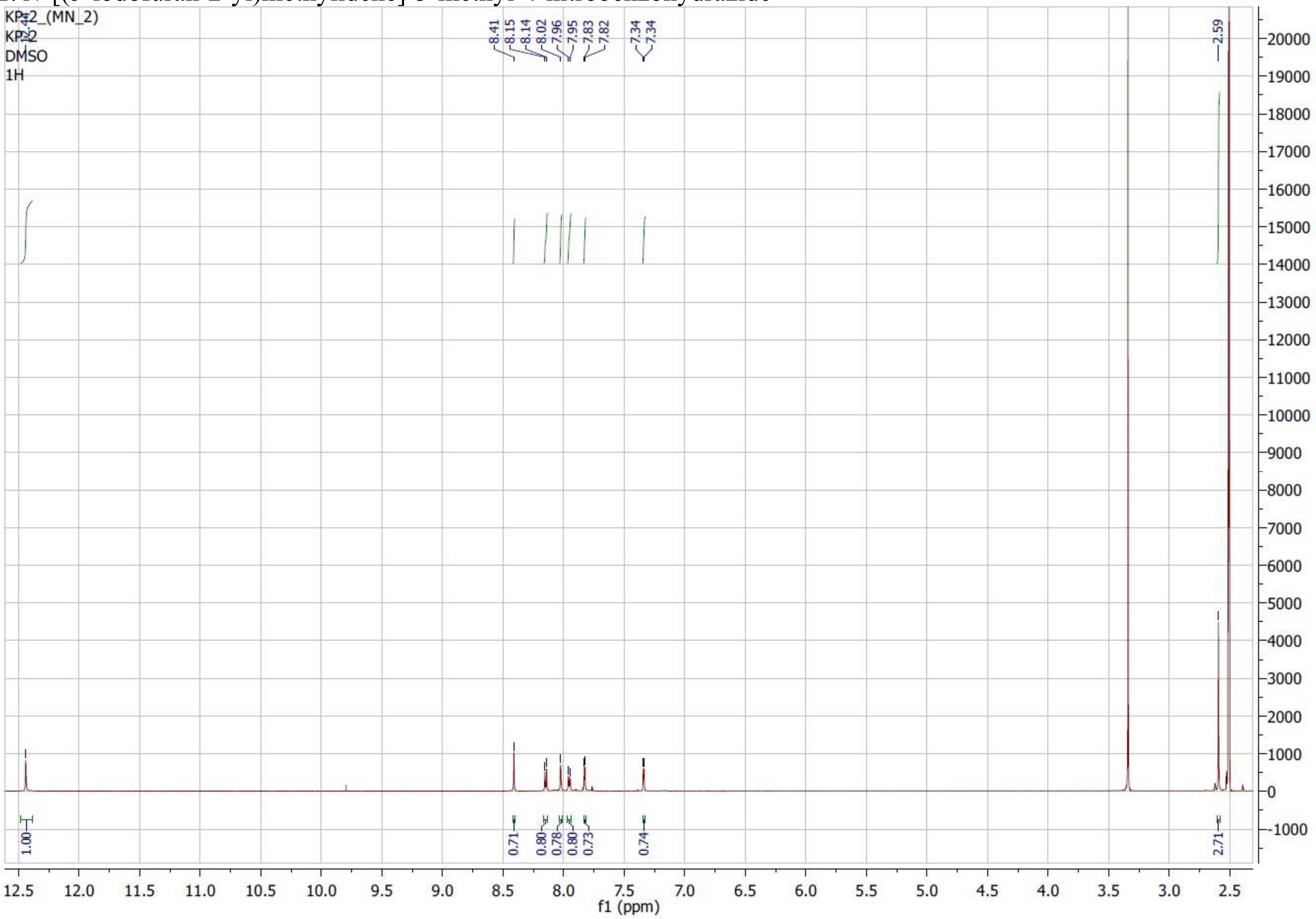

Figure S1. ^{1}H NMR spectrum of compound **2**

Compound **2**: *N*-[(5-iodofuran-2-yl)methylidene]-3-methyl-4-nitrobenzohydrazide

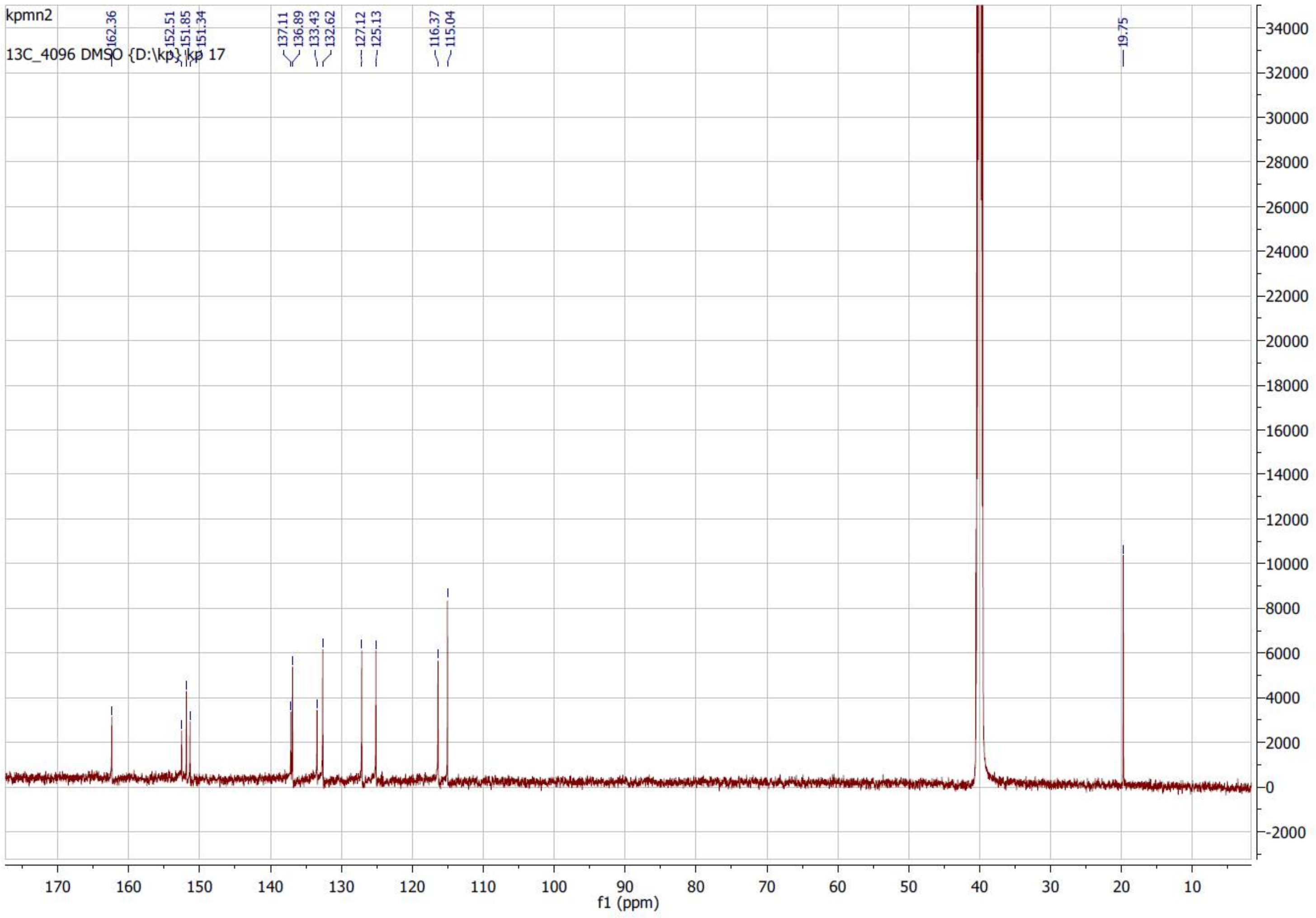

Figure S2. ^{13}C NMR spectrum of compound **2**

Compound **18**: *N*-[(5-chlorofuran-2-yl)methylidene]-3-methyl-4-nitrobenzohydrazide

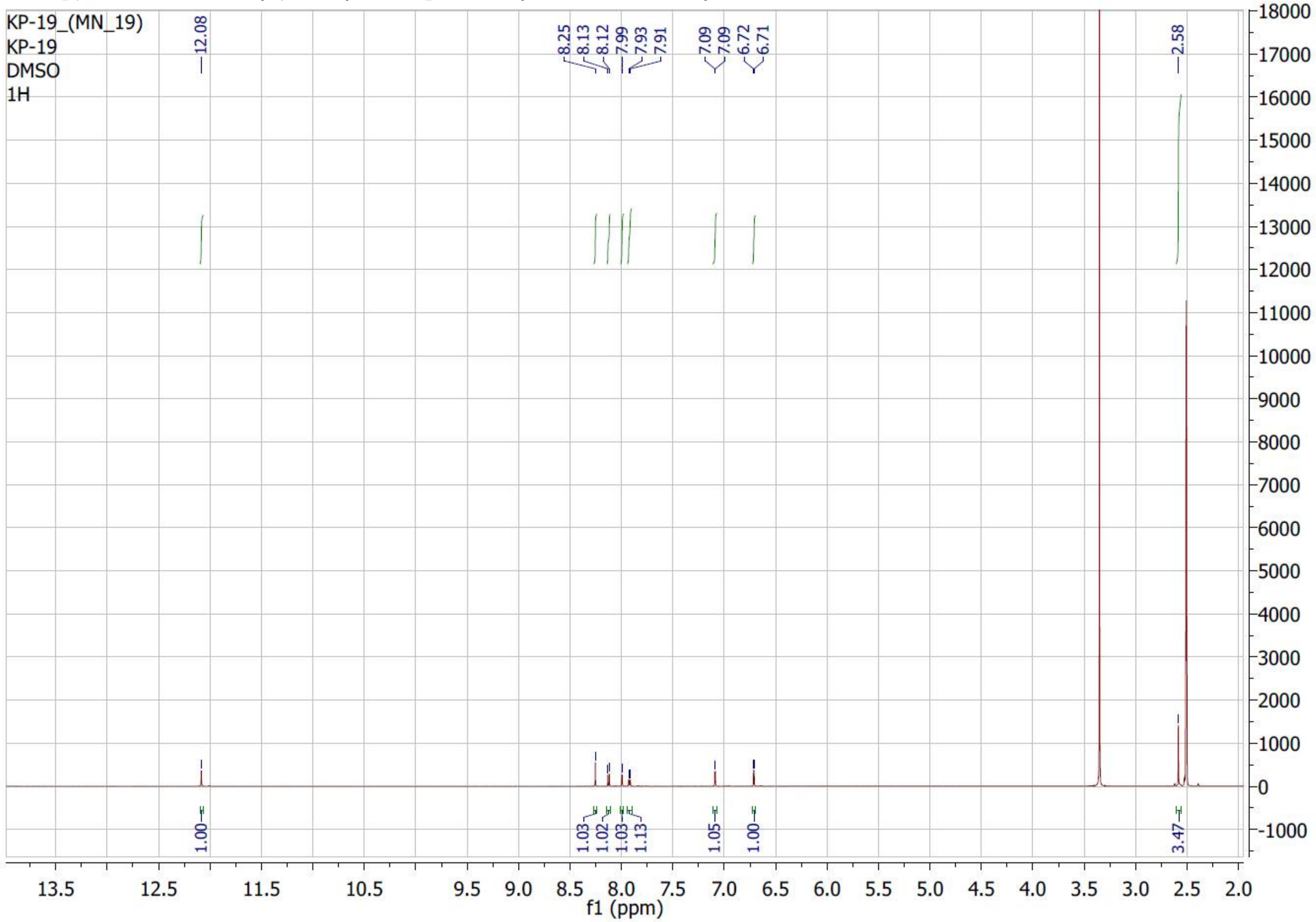

Figure S3. ^{1}H NMR spectrum of compound **18**

Compound **18**: *N*-[(5-chlorofuran-2-yl)methylidene]-3-methyl-4-nitrobenzohydrazide

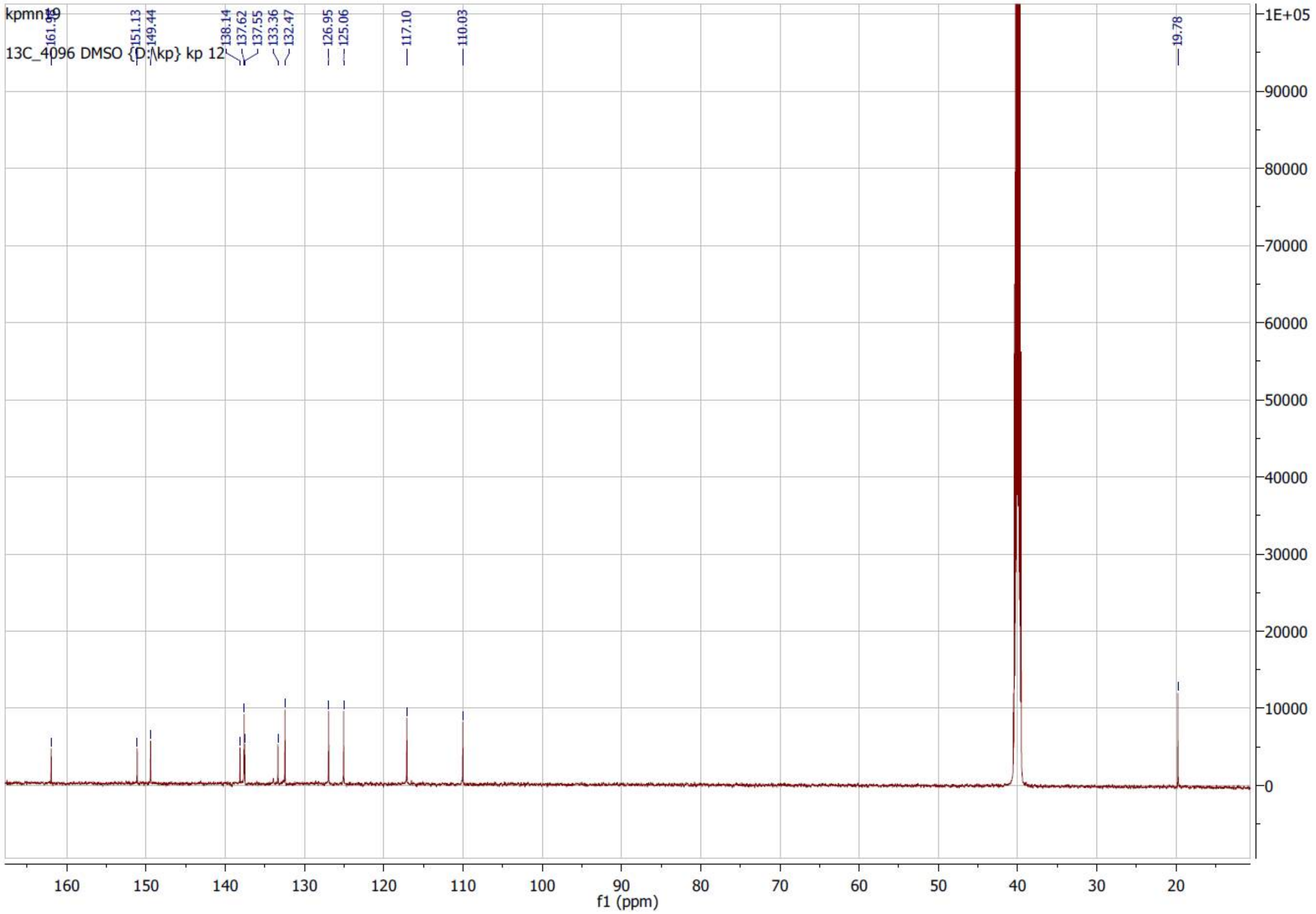

Figure S4. ^{13}C NMR spectrum of compound **18**

Compound **23**: 1-[5-(3-methyl-4-nitrophenyl)-2-(quinolin-2-yl)-1,3,4-oxadiazol-3(2*H*)-yl]ethanone

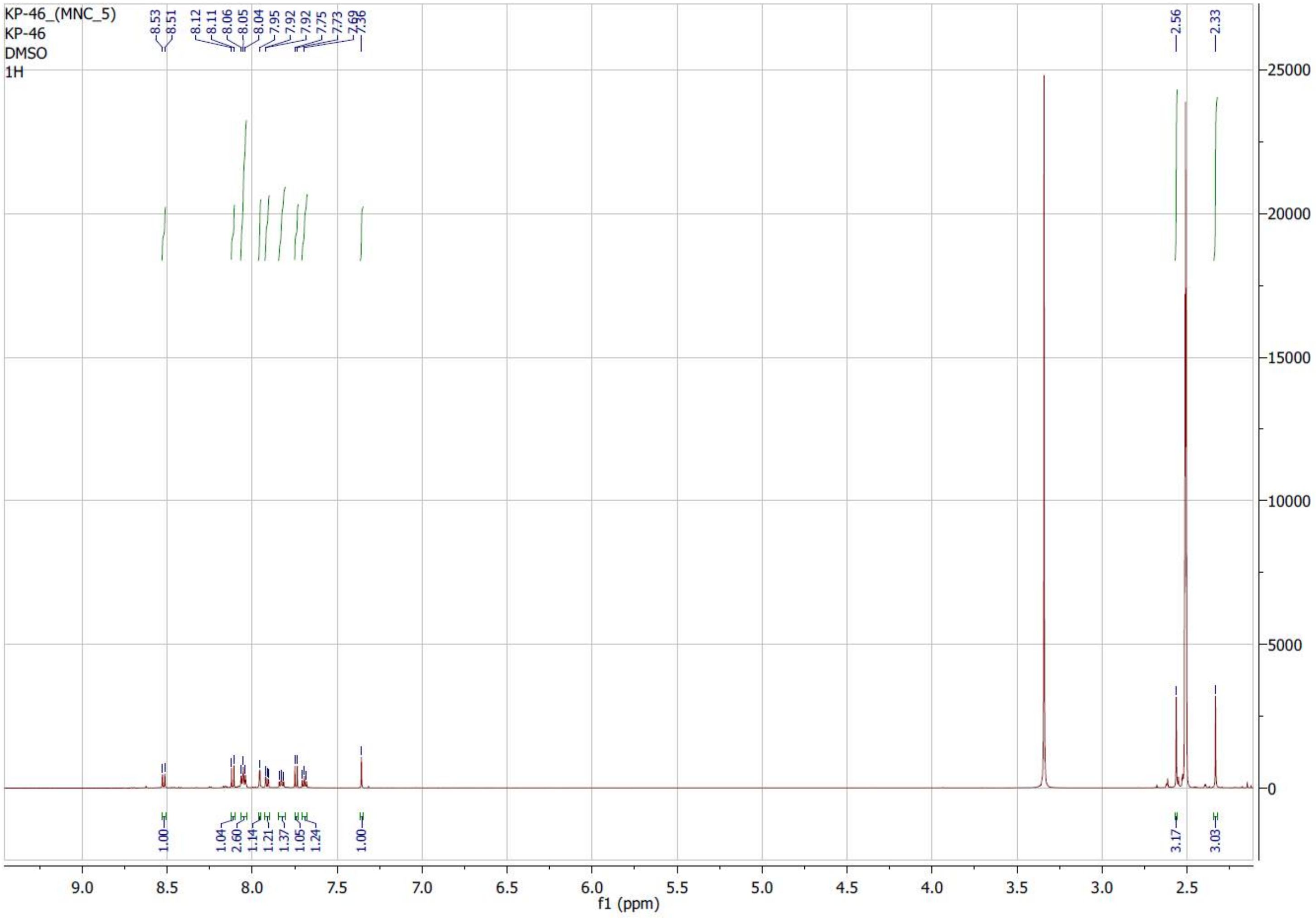

Figure S5. ^{1}H NMR spectrum of compound **23**

Compound **23**: 1-[5-(3-methyl-4-nitrophenyl)-2-(quinolin-2-yl)-1,3,4-oxadiazol-3(2*H*)-yl]ethanone

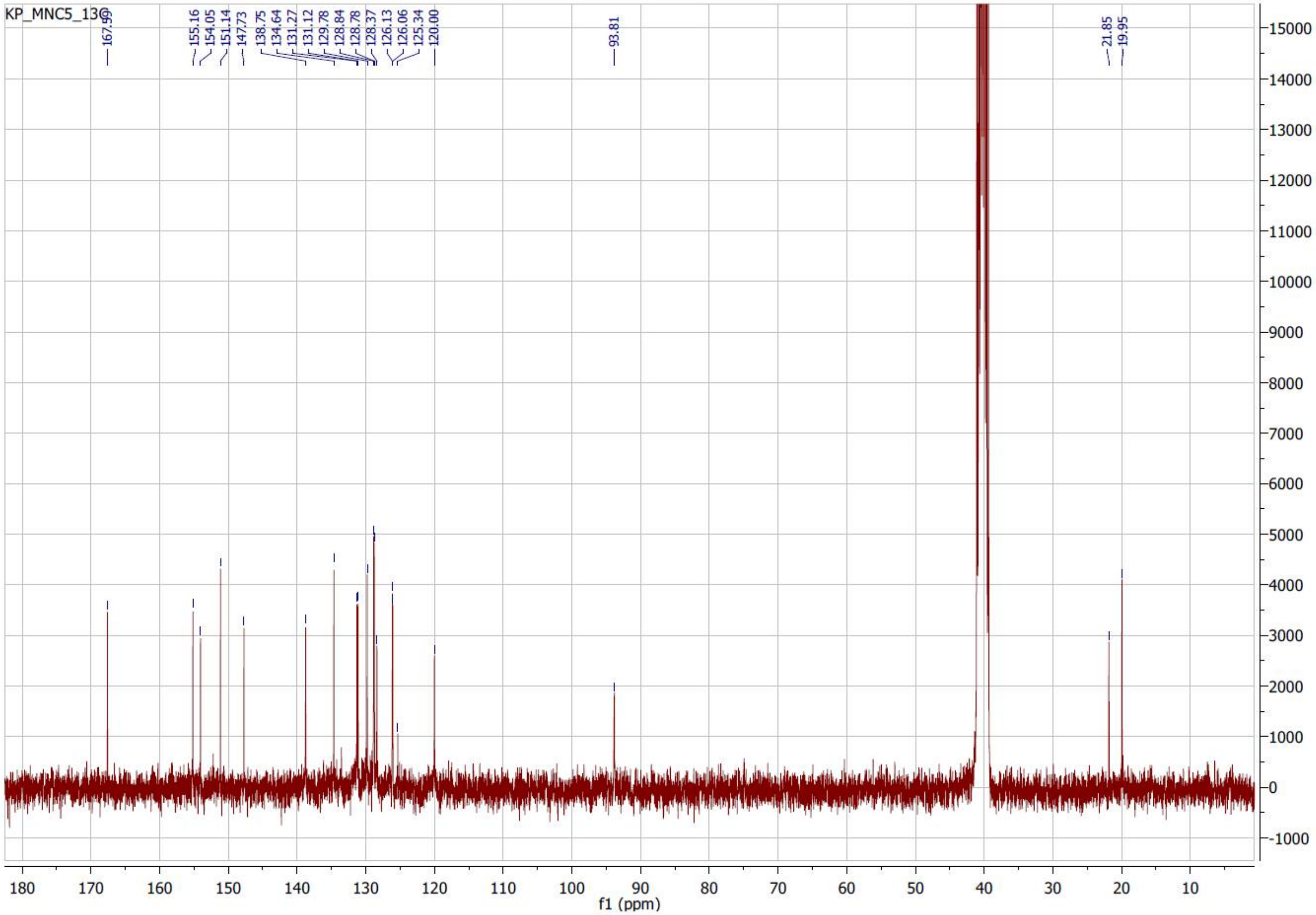

Figure S6. ^{13}C NMR spectrum of compound **23**

Compound **33**: 1-[5-(3-methyl-4-nitrophenyl)-2-(pyridin-2-yl)-1,3,4-oxadiazol-3(2*H*)-yl]ethanone

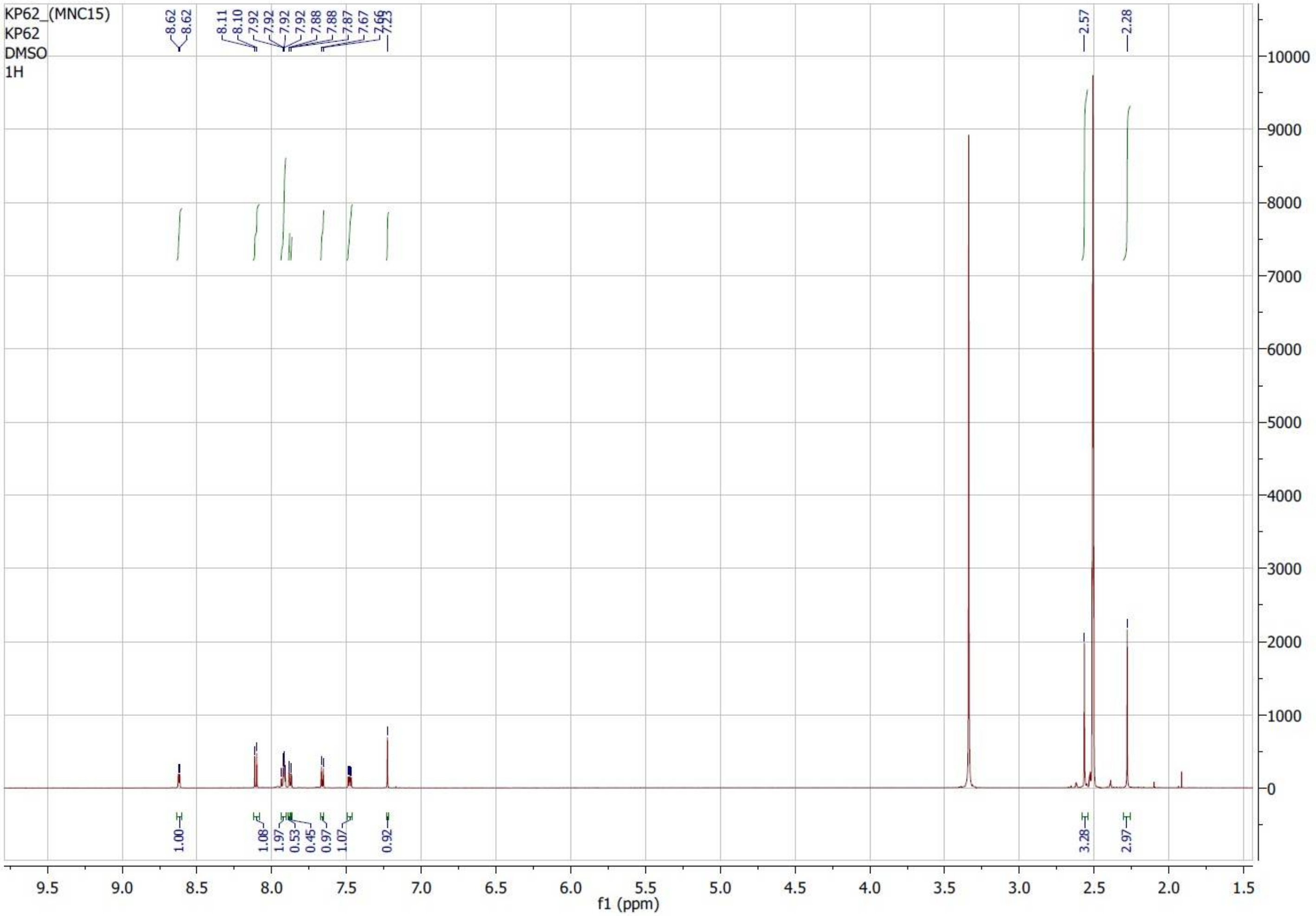

Figure S7. ^{1}H NMR spectrum of compound **33**

Compound **33**: 1-[5-(3-methyl-4-nitrophenyl)-2-(pyridin-2-yl)-1,3,4-oxadiazol-3(2*H*)-yl]ethanone

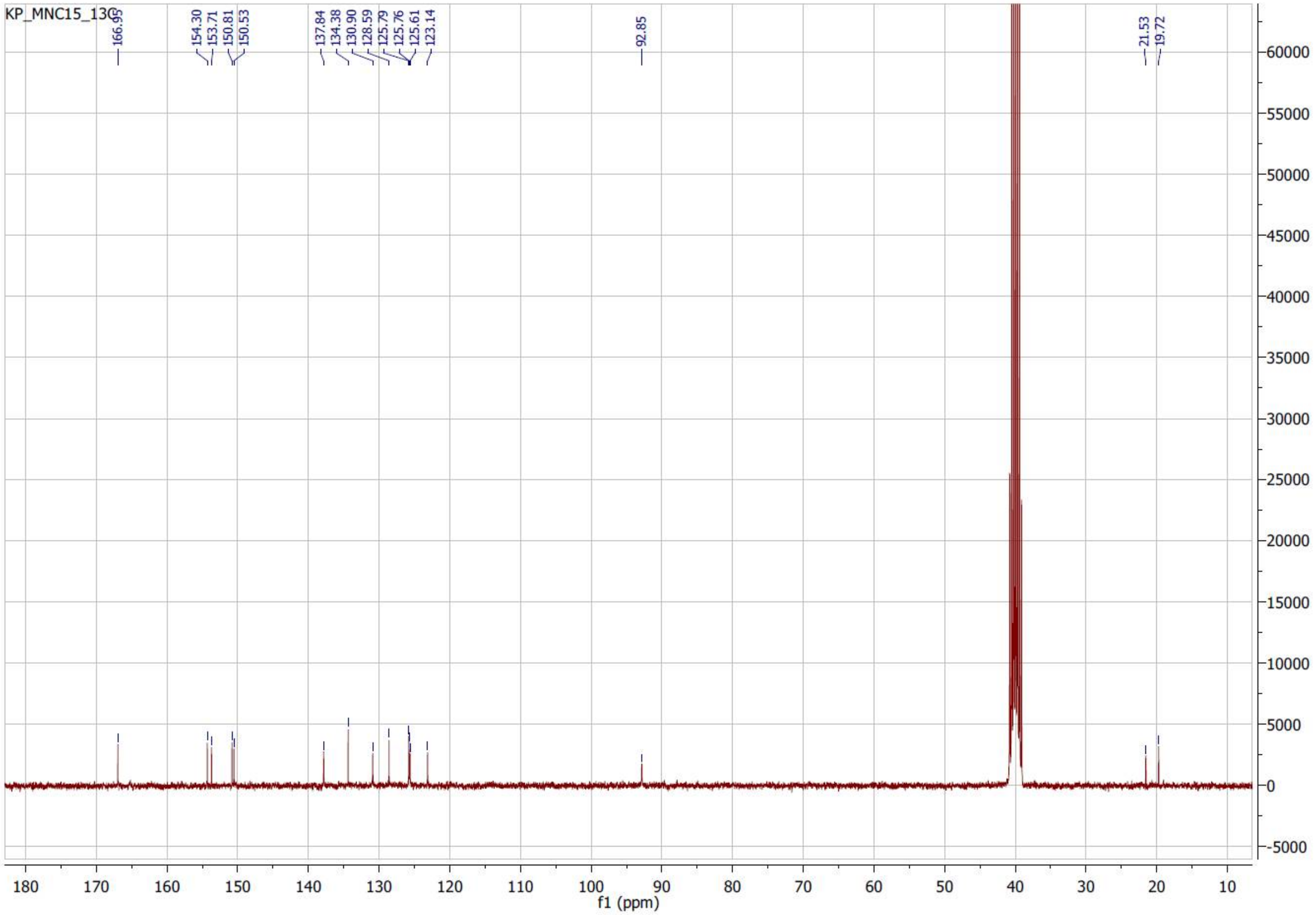

Figure S8. ^{13}C NMR spectrum of compound **33**
